# Water, not carbon, drives drought‐constraints on stem terpene defense against simulated bark beetle attack in *Pinus edulis*

**DOI:** 10.1111/nph.20218

**Published:** 2024-10-27

**Authors:** Shealyn C. Malone, R. Alex Thompson, Pak S. Chow, Celso R. de Oliveira, Simon M. Landhäusser, Diana L. Six, Katherine A. McCulloh, Henry D. Adams, Amy M. Trowbridge

**Affiliations:** ^1^ Department of Forest and Wildlife Ecology University of Wisconsin‐Madison Madison WI 53706 USA; ^2^ Department of Life and Environmental Sciences University of California‐Merced Merced CA 95343 USA; ^3^ Department of Renewable Resources University of Alberta Edmonton AB T6G 2E3 Canada; ^4^ Department of Ecosystem and Conservation Sciences University of Montana Missoula MT 59812 USA; ^5^ Department of Botany University of Wisconsin‐Madison Madison WI 53706 USA; ^6^ School of the Environment Washington State University Pullman WA 99164 USA

**Keywords:** *Ips confusus*, monoterpenes and sesquiterpenes (MST), nonstructural carbohydrates (NSC), *Ophiostoma*, phloem girdling, pressure potential, tree mortality, turgor

## Abstract

- Drought predisposes forest trees to bark beetle‐induced mortality, but the physiological mechanisms remain unclear. While drought‐induced water and carbon limitations have been implicated in defensive failure and tree susceptibility, evidence demonstrating how these factors interact is scarce.
- We withheld water from mature, potted *Pinus edulis* and subsequently applied a double‐stem girdle to inhibit carbohydrate transport from the crown and roots. Within this isolated segment we then elicited a defense response by inoculating trees with a bark beetle‐fungal symbiont (*Ophiostoma* sp.). We quantified local mono‐ and sesquiterpenes (MST), nonstructural carbohydrates (NSC), and pressure potential of the inner bark.
- Both drought‐stressed and watered trees had similar NSC concentrations just before inoculation and depleted NSC similarly following inoculation, yet MST induction (i.e. increased concentration and altered composition) was constrained only in drought‐stressed trees. Thus, NSC consumption was largely unrelated to *de novo* MST synthesis. Instead, stoichiometric calculations show that induction originated largely from stored resin. Watered trees experiencing higher pressure potentials consistently induced higher MST concentrations.
- We demonstrate the importance of preformed resin toward an induced MST response in a semi‐arid conifer where drought‐constraints on defense occurred through biophysical limitations (i.e. reduced turgor hindering resin transport) rather than through substrate limitation.

## Introduction

Climate extremes and biotic agents are common disturbances in forest ecosystems, but in recent years, the increased frequency of their incidence and interaction has driven unprecedented tree and forest mortality across the globe (Jactel *et al*., 2012; Anderegg *et al*., 2015; Kolb *et al*., 2016). Prevailing tree mortality hypotheses posit that trees become susceptible to biotic agents when drought‐induced carbon limitation constrains supply‐side processes necessary for defense metabolism (i.e. substrate availability in the form of nonstructural carbohydrates (NSC) including starch and soluble sugars) (McDowell *et al*., 2011, 2022; Huang *et al*., 2020a). However, empirical evidence directly linking water, carbon, and defense is scarce, and whether drought‐induced carbon limitation constrains tree defensive capacity remains unclear (Huang *et al*., 2020b, 2023; Erbilgin *et al*., 2021; Trowbridge *et al*., 2021). This knowledge gap may be in part because the mechanisms governing carbon reserve availability and mobilization for defense synthesis have yet to be elucidated. For example, while defense may become constrained by reduction in whole‐tree carbon reserves, defense my also become constrained by local carbon depletion (Wiley *et al*., 2016) when drought‐induced phloem failure reduces carbohydrate transport for secondary metabolite synthesis (Sala *et al*., 2010). Because phloem transport is largely driven by hydrostatic pressure generated by water from the xylem (Münch, 1930), water‐limited trees may be unable to maintain adequate phloem turgor to support the movement of carbon reserves to tissues in need (Sevanto, 2014; but see Gersony & Holbrook, 2022). Thus, trees faced with concurrent drought and biotic challenge may experience defensive failure through water × carbon interactions resulting in diminished locally available carbon reserves and impaired transport of more distally stored reserves (Salmon *et al*., 2018).

Understanding how tree water × carbon status modulates defense during drought is imperative for understanding tree resistance to bark beetles. Bark beetles attack the main stem and branches of trees where they reproduce and feed within the inner bark (Raffa *et al*., 2015). Thus, continuous access to carbon reserves may be critical for sustaining an effective defense response (Miller & Berryman, 1986; Guérard *et al*., 2007; Goodsman *et al*., 2013). In conifers, NSC provide substrate for the secondary metabolites that comprise resin, namely mono‐, sesqui‐, and diterpenes (Roth *et al*., 2018; Trowbridge *et al*., 2021). Monoterpenes have been shown to increase tree resistance against bark beetles in a dose‐ and composition‐dependent manner (Raffa *et al*., 2005, 2015), while diterpenes are most biologically active against bark beetles' fungal symbionts (Kopper *et al*., 2005); sesquiterpenes have no known activity against bark beetles or their symbionts.

**Fig. 1:**
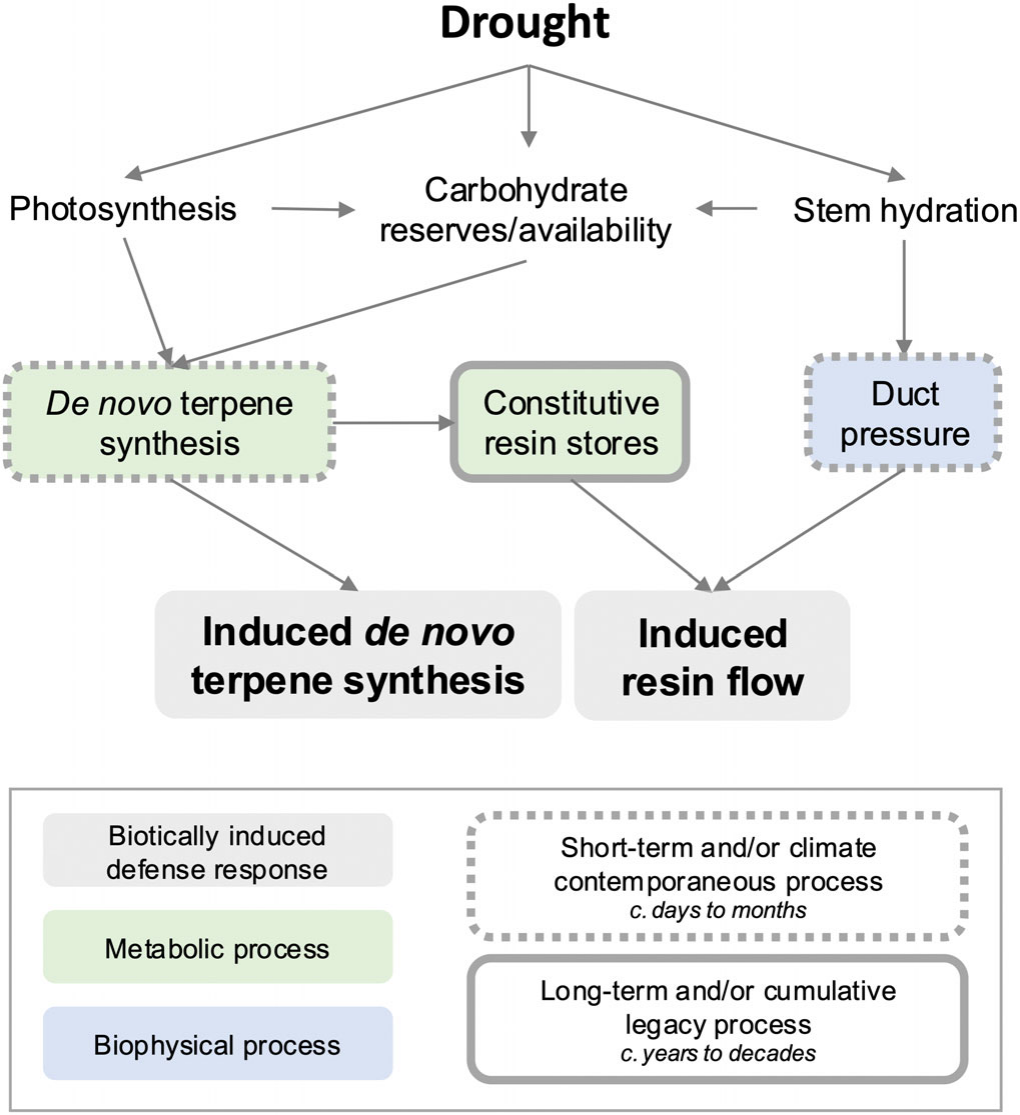
A theoretical framework demonstrating that drought's influence on conifer induced terpene defense is a dynamic process. The net effect drought on induced terpene defenses in conifers is the integration of drought's individual effects on traits and processes both metabolic and biophysical over timescales short and long. Note that the relative contribution of each induced pool (*de novo* terpene synthesis vs resin flow) toward the total induced defense response will vary by taxa, ontogeny, phenology, and so on.

Changes in conifer terpene concentration and composition following bark beetle attack, herein referred to as the ‘induced terpene response’ or ‘terpene induction’, are influenced by both preformed resin and *de novo* terpene synthesis (Fig. 1; Martin *et al*., 2002; Byun‐McKay *et al*., 2006; Chen *et al*., 2019). In the genus *Pinus*, terpene synthesis and storage occur largely in networks of interconnected resin ducts (Krokene & Nagy, 2012), which are lined with epithelial cells that synthesize and secrete terpenes into the duct lumen where they are stored under pressure long‐term (Trapp & Croteau, 2001; Zulak & Bohlmann, 2010). Upon bark beetle attack, damage to pressurized ducts causes the release of stored terpenes to the site of attack, while local *de novo* terpene synthesis rapidly upregulates in the epithelial cells promoting the production of additional terpenes in the days and weeks that follow (but note that relative and absolute increases among individual terpene compounds and/or classes may vary; Keefover‐Ring *et al*., 2016; Mason *et al*., 2017; Chen *et al*., 2019). While the exudation of preformed resin is conceptually considered an aspect of ‘constitutive’ (or existing) tree defense and *de novo* production an aspect of ‘induced’ defense (Franceschi *et al*., 2005; Krokene, 2015), measurements quantifying conifer ‘induced’ defense (e.g. resin flow or terpene concentration in response to a biotic challenger) include aspects of both (Lewinsohn *et al*., 1991). Along these lines, ‘coordination’ of constitutive and induced defenses has been observed in some *Pinus* species where defense traits like constitutive terpenes, induced terpenes, and resin flow are positively correlated (Boone *et al*., 2011; Howe *et al*., 2020, 2024). Despite two distinct sources of terpenes – stored and *de novo* – contributing to the induced response, the relative contribution of each is difficult to disentangle and is seldom accounted for when assessing the defensive capacity of individual trees.

Determining the degree to which trees rely on *de novo* vs stored terpenes can provide key insights into the physiological thresholds that constrain induced terpene defense during drought, as well as defense response variability within and across conifer taxa (Hudgins *et al*., 2004). Carbon substrate is critical to the synthesis of both *de novo‐*induced and stored resin terpenes, but on different timescales and through different processes (Fig. 1). While carbon substrate limitation may immediately influence *de novo*‐induced terpene synthesis, long‐term resin stores will not be affected since short‐term constraints on terpene metabolism minimally impact total resin storage within the stem. Therefore, *de novo*‐induced terpene synthesis may be more influenced by contemporaneous carbon reserves, while the transport and exudation of preformed resin may be more influenced by extended, antecedent carbon limitation and/or concomitant water limitation. For example, studies in *Pinus* show that tree water stress influences resin pressure and release (Vité, 1961; Neher, 1993; Lorio *et al*., 1995; Rissanen *et al*., 2021), likely through changes in epithelial cell turgor that alters the pressure exerted on resin terpenes stored within the duct cavity (Vité, 1961). Thus, for conifers relying primarily on *de novo*‐induced terpenes toward the induced response (e.g. genera like *Abies* with less substantial terpene‐storage structures), thresholds of defensive failure may be more tightly coupled with loss of phloem turgor and/or local carbon limitation; for conifers relying predominantly on stored resin for the induced response, thresholds of defensive failure may instead be more closely linked with long‐term periods spent under zero‐assimilation and/or a loss of adequate pressure within resin ducts. Such genera‐ and species‐specific patterns may help explain disparate conifer defense strategies across phylogeny and environmental gradients (Coley *et al*., 1985; Lewinsohn *et al*., 1991).

*Pinus edulis* (Engelm.), or two‐needle piñon pine, is an ecologically important and dominant tree species of the semi‐arid southwestern United States. *Pinus edulis* experienced significant and widespread mortality from 2002 to 2003 following a multi‐year drought and an unprecedented outbreak by the specialist piñon pine engraver beetle, *Ips confusus* (LeConte) (Breshears *et al*., 2005). While *I. confusus* most commonly attack larger size classes of *P. edulis* (root collar diameter (RCD) > 15 cm), they are known to attack trees with RCD as small as 5–10 cm (Negrón & Wilson, 2003; Floyd *et al*., 2009). Drought has been shown to decrease *P. edulis* carbon reserves in the crown, stem, and roots (Adams *et al*., 2013; Sevanto *et al*., 2014; Dickman *et al*., 2015; Thompson *et al*., 2023) while increasing susceptibility to attack by *I. confusus* (Gaylord *et al*., 2013). Additionally, phloem turgor collapse has been shown to proceed mortality in drought‐stressed *P. edulis* (Sevanto *et al*., 2014). Together, these observations suggest that drought may constrain the induced terpene defense response of *P. edulis* through whole‐tree and/or local carbon substrate limitation, and in turn, reduce tree resistance to *I. confusus*. NSC availability has been positively correlated with biotically induced terpene concentrations and negatively correlated with bark beetle attack in *Pinus contorta*, a more mesic‐adapted *Pinus* species (Goodsman *et al*., 2013; Wiley *et al*., 2016; Roth *et al*., 2018). Yet if and how drought‐induced limitations on carbon availability constrain bark beetle‐ × fungi‐induced terpene composition and concentrations in *Pinus* species adapted to more arid regions remains unclear. Identifying the physiological mechanisms of defensive failure in xeric‐adapted species such as *P. edulis* will be paramount for identifying species‐specific thresholds of risk from biotic agents and improving current mortality models (Breshears *et al*., 2018; Wion *et al*., 2022).

Here, reproductively mature, potted *P. edulis* trees experiencing varying levels of physiological drought stress were treated with a double‐stem girdle to manipulate phloem carbon transport from both the canopy and the roots (Miller & Berryman, 1986). We then elicited an induced terpene response by inoculating trees with an *Ophiostoma* fungus consistently isolated from field‐collected *I. confusus*. We measured local mono‐ and sesquiterpene defenses (MST), NSC (including starch, sucrose, glucose, and fructose), and pressure potential (i.e. positive pressure exerted by cells against cell walls when turgid; Bartlett *et al*., 2012) of the inner bark. We also employed stoichiometric calculations to estimate the proportion of induced MST synthesized *de novo*. Given widespread observations of declining NSC reserves in *P. edulis* during drought, we hypothesized that (H1) drought would constrain the induced MST response (i.e. reduced magnitude and altered composition) by reducing the local and distal carbon reserves available for *de novo* MST production. Specifically, we predicted that induction would be most robust in trees that were well‐watered without the girdle (access to both high‐local NSC concentrations and transported sugars) followed by well‐watered with the girdle (high‐local NSC but no transport potential), drought‐stressed without the girdle (low‐local NSC and reduced transport potential), and finally, drought‐stressed with the girdle (low‐local NSC and no transport potential). Along these lines, we expected that (H2) the induced response in *P. edulis* would be dominated by *de novo* MST synthesis, and therefore, NSC availability and MST induction would be positively correlated. Alternatively, if drought did not affect MST induction through substrate limitation for *de novo* synthesis, we expected that (H3) water status would affect induction directly through its effects on stored resin where more hydrated trees experiencing greater pressure potential (and turgor) could more effectively transport stored resin terpenes to the inoculation site.

## Materials and Methods

### Study design and physiological drought‐stress monitoring

Fifty mature *Pinus edulis* (Engelm.) (two‐needle piñon pine; *c*. 30‐yr‐old; L. Heidrich, pers. comm.) were spade dug from 60 ha of native piñon‐juniper woodland located *c*. 100 km south of Pueblo, CO (37.4856389, −104.8916389) during mid‐October 2020 (Supporting Information Fig. S1a). Trees were balled‐and‐burlaped and transported to the West Madison Agricultural Research Station (Madison, WI, USA) where they were immediately transplanted into 70 l pots with their native soil (Fig. S1b). Of the 50 trees, 20 showed visible evidence of reproductive maturity, bearing at least one cone upon arrival. However, potting alone caused trees to drop cones as they were maneuvered so we suspect that additional cones may have dropped before arrival as trees were excavated from the ground and transported cross‐country. Trees acclimated to glasshouse conditions over an eight‐month period in which they were kept well‐watered with no additional fertilizer. Soil volumetric water content (SVWC) was measured weekly (TDR probe, CS 616; Campbell Scientific, Logan, UT, USA) and trees were watered when SVWC dropped below 20%. Because of the stout stature and long‐lived crown of *P. edulis*, we removed two to three branches from the lower main stem of each tree (Fig. S1c) on 4 March 2021 to create a branchless section along the stem (*c*. 15 cm). This allowed us to apply a top girdle at the base of the live crown and bottom girdle just above the root collar of the branchless section to create an isolated stem section (hereafter, referred to as the ‘inoculation zone’) where bidirectional carbohydrate influx from the canopy and roots was prevented. Though the pruning tool was not sterilized between trees, we observed no evidence of disease or systemic damage in response to pruning during the 3‐month acclimation period. Only the trees showing evidence of a successful transplant (i.e. shoot elongation from terminal buds) at the conclusion of the acclimation period were used in the study (*n* = 46). Mean tree height and stem diameter at the root collar (RCD) were 1.35 ± 0.09 m and 7.03 ± 1.02 cm, respectively; thus, all trees fell within the observed host range size of *I. confusus* (LeConte) (RCD = 5–47 cm; Negrón & Wilson, 2003).

**Fig. 2:**
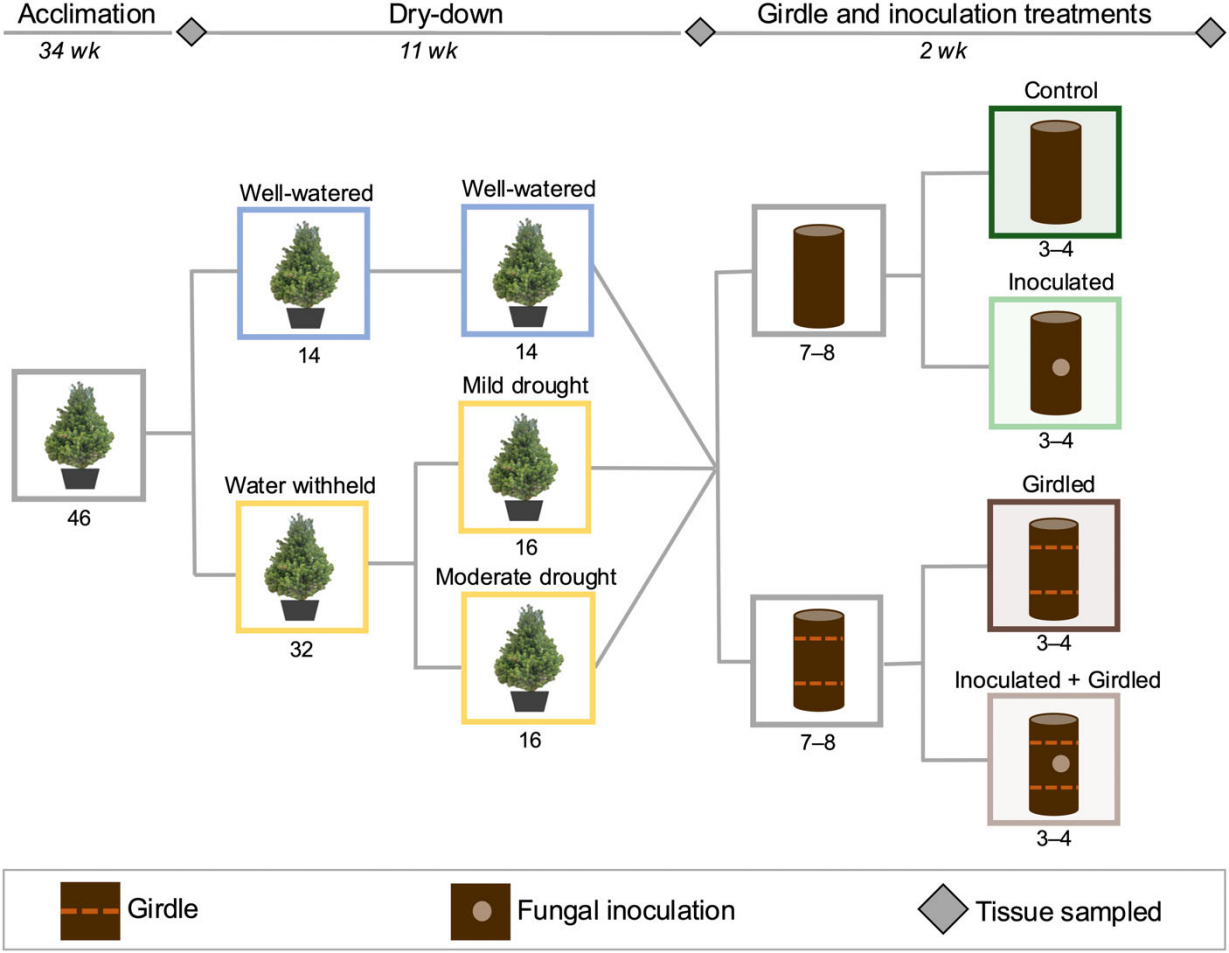
Experimental design to assess how drought, phloem connectivity, fungal inoculation, and their interaction influenced the induced mono‐ and sesquiterpene (MST) responses in *Pinus edulis*. Following glasshouse acclimation and an 11‐wk dry‐down period, mature trees were divided into three groups based on their predawn water potential: well‐watered (*n* = 14; ψ_pd_ from −0.31 to −0.91 MPa), mild physiological drought stress (*n* = 16; ψ_pd_ from −1.66 to −2.85 MPa), or moderate physiological drought stress (*n* = 16; ψ_pd_ from −2.86 to −3.61 MPa). One of four treatments was then evenly assigned across each group: control (nongirdled + noninoculated), girdled (girdled + noninoculated), inoculated (nongirdled + inoculated), or inoculated + girdled. Inner bark tissue was collected at three time periods (represented by grey diamonds): predrought, preinoculation (immediately before the application of girdle and inoculation treatments), and 2 wk postinoculation. Estimated inner bark turgor was measured preinoculation only. Water and drought treatments are represented by blue and yellow boxes, respectively. Control, inoculated, girdled, and inoculated + girdled treatments are represented by dark green, light green, dark brown, and light brown, respectively.

Following the acclimation period, we subjected trees to an 11‐wk dry‐down (Fig. 2). Trees were watered to field capacity on 18 June 2021, after which, water was withheld (*n* = 32) for the remainder of the experiment. We continued to water a subset of control trees (*n* = 14) to field capacity weekly. Though water was withheld from all drought‐stressed trees, we made use of inherent tree‐to‐tree variability (e.g. canopy and root area) to obtain a gradient of physiological drought stress across all drought‐stressed trees (as determined by predawn shoot water potential, ψ_pd_). Indeed, at the conclusion of the dry‐down, drought‐stressed trees exhibited ψ_pd_ ranging from −1.66 to −3.61 MPa. We monitored ψ_pd_ weekly to determine when trees had reached a target range of water potentials representative of key physiological thresholds related to mortality risk for *P. edulis*, but before the lethal threshold for hydraulic failure (−4.0 MPa; Koepke & Kolb, 2013; Hammond *et al*., 2019). The key physiological thresholds included (1) the point at which attack by *I. confusus* has been observed in the field (−2.3 MPa; Gaylord *et al*., 2013), (2) the zero‐assimilation point (−3.0 MPa; Thompson *et al*., 2023), and (3) the shoot turgor‐loss point (−3.1 ΜPa; Meinzer *et al*., 2014). To measure ψ_pd_, we excised distal shoots before dawn and stored them in a chilled cooler in sealed plastic bags with a damp paper towel to maintain 100% humidity. We measured shoot water potential within 1–2 h of collection using a Scholander pressure chamber (PMS Instruments, Albany, OR, USA).

Once trees reached a suitable range of water stress (−0.31 to −3.61 MPa), they were grouped according to ψ_pd_ so that girdle and inoculation treatments could be systematically assigned to individuals experiencing a range of stress (*n*_well‐watered_ = 14, *n*_mild drought_ = 16, *n*_moderate drought_ = 16). The mean water potentials of well‐watered, mild drought, and moderate drought groups were −0.62 ± 0.15, −2.35 ± 0.36, and −3.23 ± 0.21 MPa, respectively. Within each group, trees were randomly assigned to one of four treatments (*n* = 3–4): control (noninoculated and nongirdled), girdled (noninoculated and girdled), inoculated (inoculated and nongirdled), or inoculated + girdled (Fig. 2). As intended, mean ψ_pd_ did not vary among the four treatment groups (*P* = 0.9, −2.13 ± 1.11 MPa). Though we used two drought grouping to ensure that treatments were evenly distributed across individuals experiencing a range of stress (i.e. mild and moderate drought), trees were grouped as either ‘watered’ (well‐watered) or ‘drought’ (mild and moderate drought combined) for the analysis. To verify reduced assimilation and stomatal closure along the drought continuum, we measured mid‐morning net photosynthesis (*A*_net_, μmol m^−2^ s^−1^) and stomatal conductance (*g*_s_, mol m^−2^ s^−1^) throughout the drought (Fig. S2; Methods S1).

### Girdle (G) and inoculation (I) treatments

The bark girdle treatment was applied near the inoculation zone of the stem to prevent the translocation of carbohydrates from either the crown or the root system. Trees were girdled on 5 September 2021 by removing two 1‐cm bands of bark (inner and outer bark including the cambium), *c*. 10 cm apart along the circumference of the main stem (Fig. S3a). The girdles were immediately wrapped with Parafilm (Sigma‐Aldrich) to prevent xylem dehydration and contain resin flow in response to wounding. The following day, the fungal inoculation treatment was applied using plugs of agar with an active culture of *Ophiostoma* sp. previously isolated from *I. confusus* beetles collected from the Sevilleta National Wildlife Refuge, NM, USA (34.386389, −106.529444). We removed two adjacent 6 mm sections of the outer and inner bark tissue centered in the isolated stem section using a metal punch *c*. 12.3 ± 0.6 cm above the root collar. To control for the injury response of inoculation, bark plugs were immediately reinserted for noninoculated trees, while for inoculated trees we applied a 5 mm plug of agar with actively growing *Ophiostoma* sp. mycelium before replacing each bark plug (Fig. S3b). In all cases, we wrapped the inoculation site in Parafilm for 24 h to prevent desiccation and contamination.

### Quantification of mono‐ and sesquiterpenes (MST) and nonstructural carbohydrates (NSC)

To determine the effect of drought and phloem connectivity on the induced MST response and NSC concentrations, we collected inner bark tissue at three times: predrought (immediately before the onset of the drought treatment on 14 June 2021), preinoculation (after 11 wk of drought, but immediately before girdling + fungal inoculation on 3 September 2021) and postinoculation (2 wk after fungal inoculation on 20 September 2021) (Fig. 2). Tissue sampled predrought and preinoculation was collected from two 10 mm bark punches taken on separate sides of the isolated stem segment (Fig. S3c). Tissue sampled 2 wk postinoculation were collected using a scalpel to ensure that samples included tissue directly surrounding the two inoculation punches (*c*. 3 × 5 cm; Fig. S3d). Special care was taken to reduce the effect of wounding from one collection site on another. Specifically, the three collection sites were slightly staggered in height and evenly spaced around the circumference of the inoculation zone. Excised tissue samples were immediately flash frozen in liquid nitrogen and transported to the University of Wisconsin–Madison where samples were stored at −80°C before MST and NSC extraction.

To prepare inner bark tissue for metabolite extraction, inner bark was separated from the outer bark, diced into *c*. 1 mm^2^ cubes, and partitioned into two subsamples for quantification of MST and NSC. Tissue used to determine MST content was immediately submerged in dimethyl chloride while tissue used to determine NSC content was microwaved for 180 s and oven‐dried at 70°C before being shipped to the University of Alberta for extraction and quantification. MST were extracted and quantified by gas chromatography–mass spectroscopy (GC‐MS) according to Trowbridge *et al*. (2021). NSC (starch, sucrose, glucose, and fructose) were extracted and quantified photometrically following the standard enzymatic methods (Landhäusser *et al*., 2018). Details are described in Methods S2. We were unable to separate the inner bark of four trees sampled postinoculation because the tissue was desiccated and paper‐thin, likely in response to the drought treatment. Therefore, our postinoculation analysis includes responses from the 42 trees from which inner bark tissue was available: control (*n*_well‐watered_ = 3, *n*_drought_ = 8), girdled (*n*_well‐watered_ = 3, *n*_drought_ = 6), inoculated (*n*_well‐watered_ = 4, *n*_drought_ = 7), or inoculated + girdled (*n*_well‐watered_ = 4, *n*_drought_ = 7).

### Correction for girdle‐induced resin loss from inoculated and girdled (I + G) trees

Trees were girdled to disrupt the flow of carbohydrate reserves to the inoculation zone, and indeed, the girdle treatment was successful in doing so (see the Results section). However, when comparing inner bark MST concentrations among treatments, it became apparent that the girdle alone caused a slight but significant reduction in the inner bark MST during the 2‐wk inoculation period compared to control trees (Fig. S4), likely due to the resin loss from mechanical wounding. This unintended treatment effect made it difficult to isolate the contribution of local vs distal carbohydrates to MST synthesis because the girdle directly reduced terpene concentrations via resin loss. Therefore, we corrected for girdle‐induced resin loss in inoculated + girdled trees by adding back the relative concentration of MST lost due to the girdle. To calculate the relative concentration of girdle‐induced resin loss, we took trees within the girdle alone treatment (G) and subtracted preinoculation MST concentration from postinoculation concentration and divided the difference by preinoculation levels. Next, we accounted for the effects of drought on girdle‐induced resin loss (watered trees tended to have greater resin loss than those that were drought‐stressed) by creating a linear model with ψ_pd_ and relative MST loss as the predictor and response variable, respectively (Fig. S5). We then used this model to predict the relative amount of girdle‐induced MST loss in inoculated + girdled trees based on each individual tree's ψ_pd_ (correction values ranged from 0, no resin loss to −0.43, 43% resin loss); this value was then used to calculate the ‘girdle‐corrected’ induced MST concentration for inoculated + girdled trees:

$$\Delta T_{\text{corrected}}=\Delta T_{\mathrm{raw}}+\left(\Delta T_{\mathrm{raw}}\times \left|\text{Correction}\right|\right)$$

where $\Delta T_{\mathrm{raw}}$ is the difference between post‐ and preinoculation MST concentration, Correction is the relative MST loss based on ψ_pd_ (predicted from trees that were girdled only), and $\Delta T_{\text{corrected}}$ is the girdle‐corrected induced change in MST concentration (ΔMST, mg g^−1^) for inoculated + girdled trees. A positive change in $\Delta T_{\text{corrected}}$ was interpreted as MST induction in our results. All results are reported as girdled corrected values for inoculated + girdled trees ($\Delta T_{\text{corrected}}$). However, we note here that the relationships of MST induction with either NSC concentration, pressure potential, or water potential (see the Results section) were similar whether $\Delta T_{\text{corrected}}$ or $\Delta T_{\mathrm{raw}}$ concentrations were used.

### Stoichiometric calculations predicting maximum *de novo* MST synthesis potential based on local NSC for I + G trees

To estimate the maximum potential synthesis of *de novo* MST of girdled trees (I + G) based local NSC reserves alone, we used a stoichiometric approach described by Thompson *et al*. (2024). This approach employs an energetic accounting procedure that quantifies all synthesis costs related to the carbon skeleton, ATP, and reducing agents involved in MST synthesis (De Vries, 1974; Gershenzon *et al*., 1993; Banerjee & Sharkey, 2014). Based on this calculation, we estimated that 1 g of MST requires 3.34 g glucose for synthesis (see Methods S3 for details). The use of only girdled trees (I + G) for this estimate ensured that only local NSC were available as substrate and eliminates the confounding influx of distally transported NSC from the crown or the root system.

To quantify the amount of local NSC available for MST induction, we first used the mean percent of NSC depleted by girdled trees (G) to estimate the percent of NSC that were used to maintain metabolic activities (e.g. respiration) under baseline conditions (NSC_maintenance_) when trees were not inoculated. We then assumed that all NSC remaining were potentially available for MST synthesis. That is, for each I + G tree:

$${\mathrm{NSC}}_{\text{available}}={\mathrm{NSC}}_{\mathrm{pre}‐\text{inoculation}}\times \left(100-\%{\mathrm{NSC}}_{\text{maintenance}}\right)$$

where ${\mathrm{NSC}}_{\mathrm{pre}‐\text{inoculation}}$ is the total concentration of local NSC preinoculation, $\%{\mathrm{NSC}}_{\text{maintenance}}$ is the mean percent reduction in local NSC for girdled trees (52%, see the Results section), and ${\mathrm{NSC}}_{\text{available}}$ is the remaining amount of preinoculation NSC estimated available for MST synthesis. After using the glucose cost (3.34 g glucose per 1 g MST) to calculate the maximum potential MST synthesis possible (mg g^−1^) based on ${\mathrm{NSC}}_{\text{available}}$, we then calculated the maximum potential percent of induction by dividing the calculated potential MST maximum by the MST induction observed. Maximum potential MST synthesis was calculated only for inoculated + girdled trees showing evidence of induction in response to inoculation (*n* = 7), and we excluded trees that did not (*n* = 3, [MST_postinoculation_] < [MST_preinoculation_]).

### Estimated inner bark pressure potential

We estimated the mean pressure potential (ψ_pressure_) of cells of the inner bark by calculating the difference between water potential and solute potential (ψ_pressure_ = ψ − ψ_solute_) (Bartlett *et al*., 2012; Sapes *et al*., 2021; Gersony & Holbrook, 2022). Inner bark water potential (ψ) was estimated by measuring shoot ψ_pd_ (MPa, described above) while solute potential (ψ_solute_) was determined by measuring the osmolality of the inner bark tissue (mmol kg^−1^) using a Vapro 5800 vapor pressure osmometer (Wescor Inc., Logan, UT, USA). On 2–4 September 2021, immediately before girdling and inoculation treatments, we excised 6 mm bark punches from the stem of each tree *c*. 50 cm above the root collar and immediately flash froze the bark punch in several layers of aluminum foil to limit evaporation. Osmolality was measured within 10 h of tissue collection and tissue remained submerged in liquid nitrogen until measurement. Frozen inner bark discs were sliced to a thickness just under 1 mm, pierced 12–15 times to facilitate equilibration within the chamber, and then promptly sealed in the osmometer chamber for measurement. Solute potential was calculated as follows:

$$\psi_{\text{solute}}=-\frac{\text{mOsm}}{\mathrm{kg}}\times 0.0000083144598\times K$$

where $\frac{\text{mOsm}}{\mathrm{kg}}$ is osmolality and K is the chamber temperature (mean temperature was 305.0 ± 0.2 K).

### Statistical analysis

To determine the effect of physiological drought stress on the induced MST response, we fit linear models with change in MST concentration as the response variable and ψ_pd_, inoculation treatment, and their interaction as predictor variables. Change in MST concentration (hereafter referred to as ‘ΔMST’) in noninoculated trees (C and G) and ‘induced MST concentration’ in inoculated trees (I and I + G) was calculated by subtracting inner bark MST concentrations postinoculation from those measured preinoculation. To assess the effects of drought, inoculation, and their interaction on differences in MST composition, we employed permutational multivariate ANOVA (PERMANOVA; Anderson, 2014; Oksanen *et al*., 2020). We used the ‘vegdist’ function in the vegan package (Oksanen *et al*., 2020) to calculate dissimilarities among samples using the Bray–Curtis metric, and we used nonmetric multidimensional scaling (NMDS) to visualize differences among treatments. To determine the relative contribution of local vs distal NSC toward the induced MST response during drought, we fit linear models with induced MST concentration as the response variable and drought, girdle, and their interactions as the predictors.

To assess NSC dynamics in response to drought, inoculation, and girdle treatments, we fit linear models with local NSC concentration (either total NSC, starch, total sugar, sucrose, glucose, or fructose) as the response variable and drought, inoculation, girdle, and their interactions as the predictors. When predictor variables accounted for variation in the model, as determined by type II ANOVA, *post hoc* pairwise comparisons were assessed using Tukey‐HSD using the ‘emmeans’ function (Lenth, 2022). To determine the amount of variation local NSC explained with regard to total induced MST concentrations, we fit linear models with induced MST concentration as the response variable and preinoculation NSC concentration (either total NSC, starch, total sugar, sucrose, glucose, and fructose) as the predictor variable. To further explore the relationship of MST and NSC dynamics, we also fit linear models with induced MST concentration as the response variable and the absolute or relative changes in local NSC as the predictor variables. Absolute change was determined by subtracting postinoculation from preinoculation concentrations; relative change was calculated by dividing the absolute change by preinoculation concentrations.

To compare observed MST induction and potential MST *de novo* induction for inoculated + girdled trees (I + G) across drought treatment, we fit linear models with MST induction as the response variable and drought treatment, MST type (actual or observed), and their interaction as the predictor variable. *Post hoc* pairwise comparisons were assessed using Tukey‐HSD via ‘emmeans’. We also fit linear models with maximum potential percent induction as the response variable and ψ_pd_ as the predictor variable. Finally, to determine the amount of MST induction variation explained by estimated inner bark ψ_pressure_, we fit linear models with MST induction as the response variable and estimated ψ_pressure_ as the predictor variable. For all the models described above, residuals were assessed to confirm normality and homoscedasticity using diagnostic plots and Shapiro–Wilks tests.

## Results

### Drought stress reduces the magnitude of the induced MST response and alters composition

**Fig. 3:**
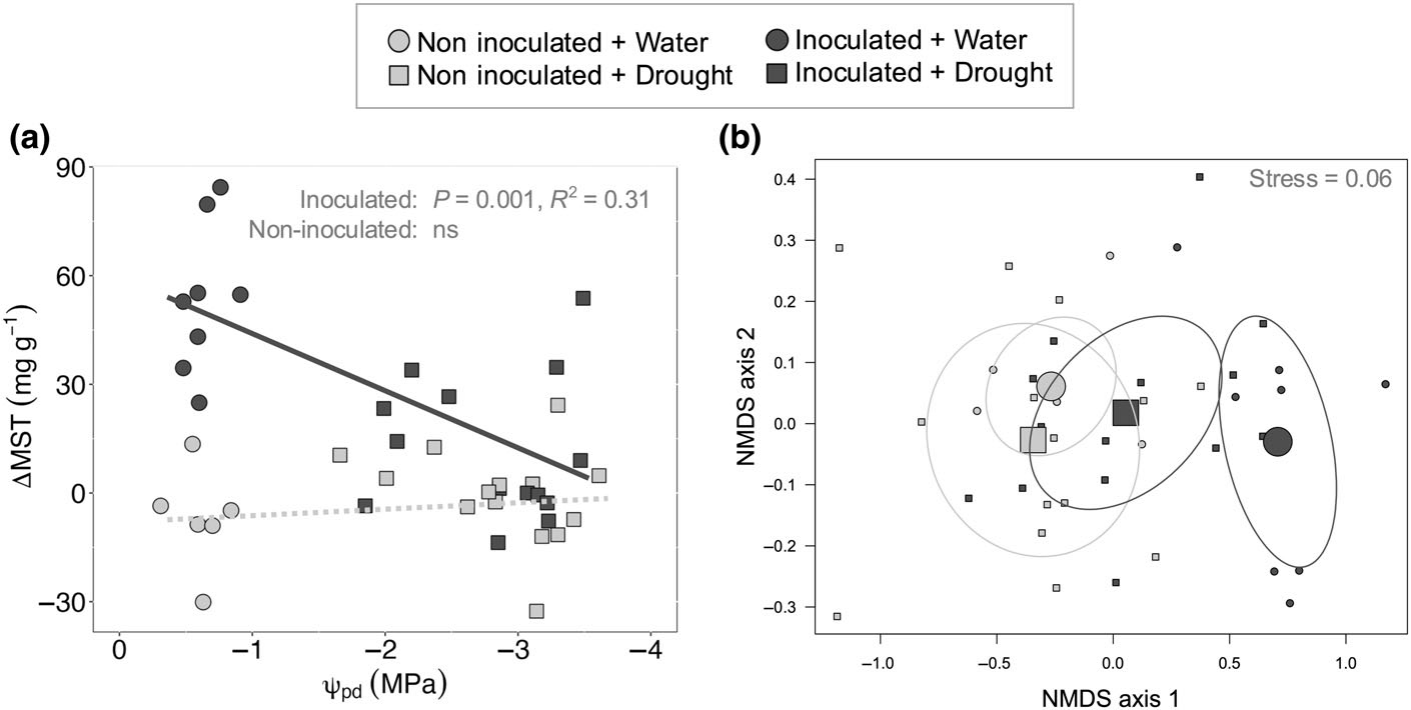
Drought alters the magnitude (a) and composition (b) of induced mono‐ and sesquiterpene (MST) in *Pinus edulis*. Relationship of inner bark MST concentration with predawn water potential (ψ_pd_, MPa) for inoculated (dark gray, *P* = 0.001) and noninoculated (light gray, ns) trees (a). Change in MST concentration (ΔMST, mg g^−1^) is the difference between post‐ and preinoculation. Noninoculated trees include those assigned to the control and girdled treatments; inoculated trees include those assigned to the inoculated and inoculated + girdled treatments. Results are from linear regression. Nonmetric multidimensional scaling (NMDS) of terpene composition in response to the interaction of drought and inoculation (b). Trees were either watered (circles) or drought‐stressed (squares) and inoculated (dark gray) or noninoculated (light gray). Ellipses encircle the centroids (large shapes) and the relative composition of each individual tree (small shapes) from the same treatment. ns, no significant difference.

A total of 43 terpene compounds were quantified using GC‐MS analysis, including 15 nonoxygenated monoterpenes, one oxygenated monoterpene, four monoterpene esters, 21 sesquiterpenes, and two oxygenated sesquiterpenes (Table S1). While drought stress alone did not affect MST concentrations, fungi‐induced terpene concentrations decreased with increasing drought stress (Fig. 3a; *P* = 0.001). Specifically, for every 1 MPa reduction in ψ_pd_, induced MST were reduced by 15.9 mg g^−1^. Similarly, drought also affected induced MST composition (i.e. the relative abundance of individual terpene compounds, Fig. 3b; *P* = 0.01). While the induced MST profile for well‐watered trees was distinctly different from the constitutive blend, drought overrode such induced shifts resulting in drought‐stressed trees exhibiting MST profiles similar to noninduced, well‐watered trees (Fig. 3b; Table S1).

### Induced MST are generally unaffected by spatial carbohydrate supply

**Fig. 4:**
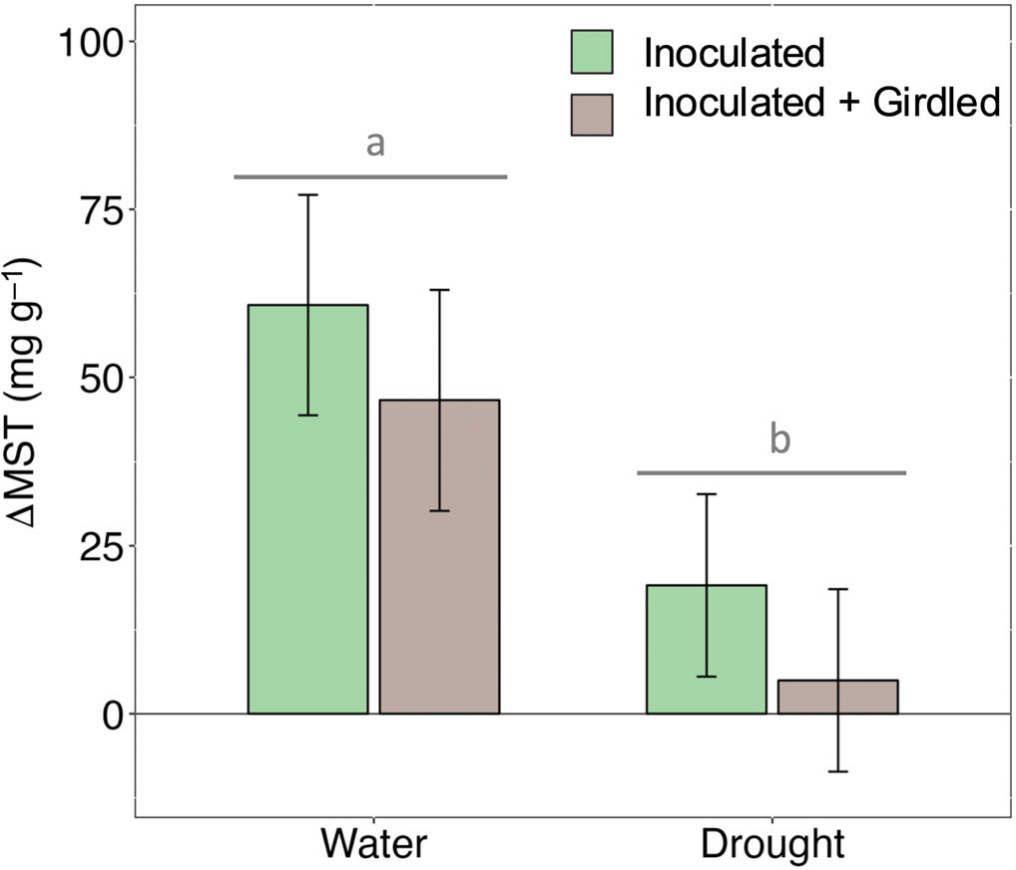
Induced mono‐ and sesquiterpene (MST) concentration was lower in drought‐stressed *Pinus edulis* and unaffected by the girdle. Induced MST concentration (ΔMST, mg g^−1^) is the difference between post‐ and preinoculation. Model means are reported across girdle treatments. Note that the girdle treatment had no effect on MST induction (*P* = 0.1, ANOVA). Error bars represent ±SE and significant differences among drought treatment are shown with gray lowercase letters (*P* < 0.05, ANOVA).

To assess the relative importance of local vs distal carbohydrate reserve availability for the induced MST response during drought stress, we applied a fungal inoculation treatment to girdled (no distal NSC availability) and nongirdled (potential access to distal NSC reserves) trees. While the girdle treatment generally reduced induced MST levels in watered and droughted trees (Fig. 4), the effect was only weakly significant (*P* = 0.1), as was the interaction (*P* = 0.1). Instead, drought alone had the greatest effect on the induced MST response (Fig. 4; *P* = 0.0001) reducing the response by almost 80% to that observed in watered trees.

### Inhibited phloem connectivity and fungal challenge together greatly reduce local NSC

**Fig. 5:**
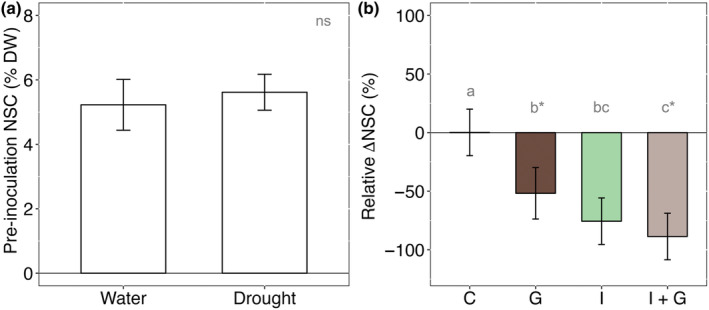
Nonstructural carbohydrate (NSC) dynamics in *Pinus edulis* in response to drought and girdle × inoculation treatments. NSC is the sum of starch, sucrose, glucose, and fructose. Inner bark NSC (% DW) did not vary among watered or drought‐stressed trees immediately before the application of the fungal and girdle treatments (a); therefore, all trees had similar concentrations of local NSC pools from which induced terpenes could be synthesized. Girdle and inoculation treatments, and their interaction, reduced inner bark NSC independent of drought (b). Relative change (Δ) in NSC (%) for an individual tree is the difference between post‐ and preinoculation concentrations divided by preinoculation concentration multiplied by 100. Treatments are control (C), girdle (G), inoculated (I), and inoculated + girdled (I + G). Error bars represent ±SE and significant differences among drought treatment are shown with gray lowercase letters (*P* < 0.05, except when significance letters are noted with an asterisk (*) in which case the comparison is marginally significant (*P* = 0.06), Tukey‐HSD following ANOVA). ns, no significant difference.

Drought alone failed to alter total NSC levels before girdling and inoculation (Fig. 5a), and this observation was driven by soluble sugars which represented 98% of the NSC pool in the inner bark across all trees (Fig. S6a; 5.4 ± 1.4% DW). Drought did, however, cause a near‐complete depletion of starch (Fig. S6b; *P* < 0.0001; 0.03 ± 0.03% DW), but even well‐watered trees maintained low‐starch concentrations in the inner bark (0.3 ± 0.1% DW).

The girdle, inoculation, and their interaction reduced relative NSC concentrations by 52 ± 11%, 76 ± 10%, and 89 ± 10%, respectively (Fig. 5b). On average, across all inoculated trees (I and I + G), fungal inoculation depleted NSC by 82 ± 7%. These results were driven by decreases in glucose and fructose (Fig. S7). On average, inoculated trees (I and I + G) had < 1% DW total NSC postinoculation while noninoculated trees (C and G) had 4% DW. Given near‐zero starch concentrations preinoculation (particularly for trees experiencing drought), we could not accurately assess changes in starch in response to the girdle and inoculation.

### Local NSC concentration did not predict MST induction and failed to support calculated levels of *de novo* MST synthesis

**Fig. 6:**
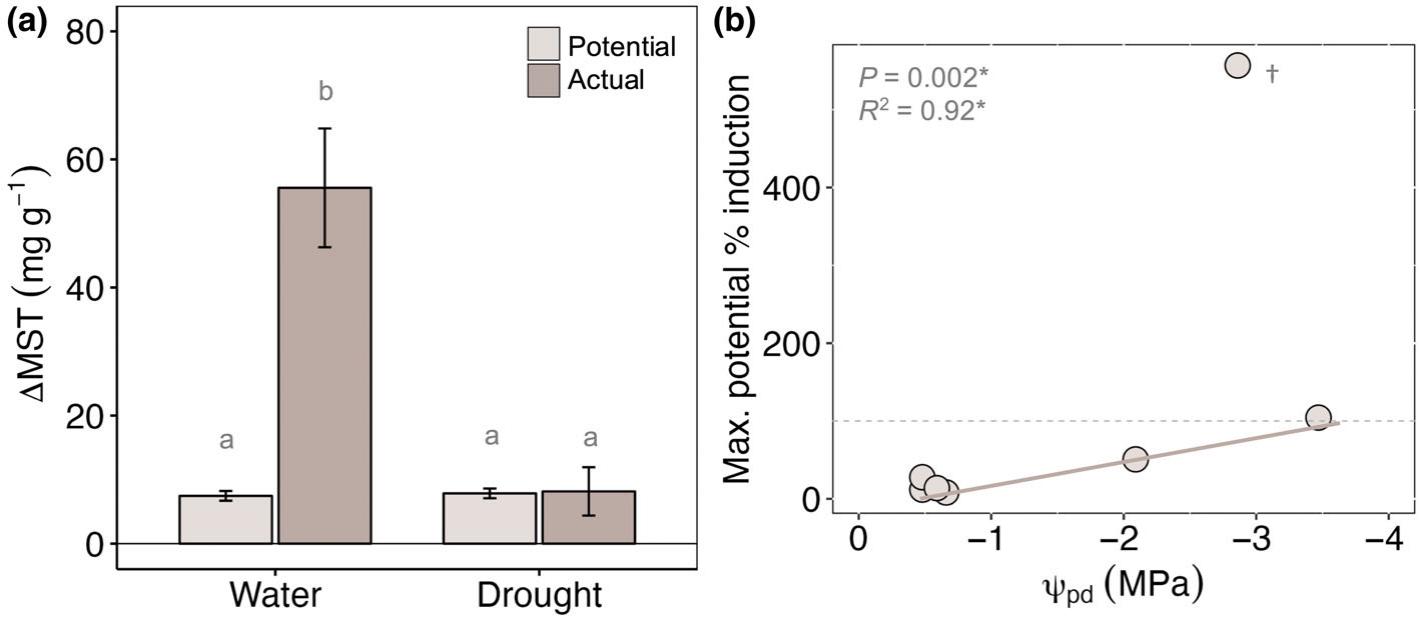
*De novo* mono‐ and sesquiterpene (MST) synthesis represents only a portion of the induced MST response in *Pinus edulis*. Maximum potential *de novo* MST synthesis (light brown) that could be supported by NSC available preinoculation compared to the actual MST induction observed (medium brown) for I + G trees in response to water availability (a; ΔMST, mg g^−1^). Note that only girdled trees (I + G) were used to estimate *de novo* MST induction to control for contributions of local NSC only. Maximum potentials were calculated assuming that 1 g MST requires 3.54 g glucose for synthesis and that 48% of preinoculation NSC concentrations were used for *de novo* MST synthesis (while the other 52% was used for maintenance respiration). Error bars represent ±SE and significant differences among groups are shown with gray lowercase letters (*P* < 0.05, Tukey‐HSD following ANOVA). Maximum potential percent induction supported by *de novo* synthesis from available NSC in response to physiological drought stress (b; ψ_pd_, MPa). Dashed gray line is the threshold above which 100% of the induced response could potentially be supported through *de novo* synthesis using NSC available preinoculation. *, *P*‐value (ANOVA) and marginal *R* ^2^ reported are for models excluding an outlier (noted with a dagger) where the maximum potential percent induction was > 500%. The outlier is shown to illustrate that drought‐stressed trees experiencing ψ_pd_ > −2.5 MPa could have induced more MST than they did based on NSC availability alone.

Preinoculation NSC concentrations did not predict the induced MST response (Fig. S8). This was true whether we assessed MST as a function of absolute or relative change in NSC (including total NSC, starch, total sugar, sucrose, glucose, and fructose; Table S2). Stoichiometric calculations showed watered trees that were girdled (i.e. access to local NSC only) exhibited induced MST concentrations nearly seven times greater than what could be supported by *de novo* synthesis from local NSC availability alone (Fig. 6a; *P* = 0.002). In other words, NSC could only account for 15 ± 10% of the actual induction observed in watered trees. Across drought treatments, only two of the seven girdled trees possessed local NSC reserves to sufficiently support the MST concentrations observed (Fig. 6b). These trees could have induced maximum potential terpene concentrations 104% and 557% greater than actual concentrations, but actual concentrations were exceedingly low (9.0 and 1.3 mg g^−1^, respectively). The estimates reported here were calculated assuming that 48% preinoculation NSC were available for defense synthesis (see the Materials and Methods section), but treatment effects were similar even when we assumed that 100% preinoculation NSC were available for defense synthesis (see Methods S4 for results). Taken together, these estimates highlight that *de novo* terpene synthesis contributes a relatively small proportion to the induced MST response in *P. edulis*.

### MST induction was constrained with decreasing water and pressure potential

**Fig. 7:**
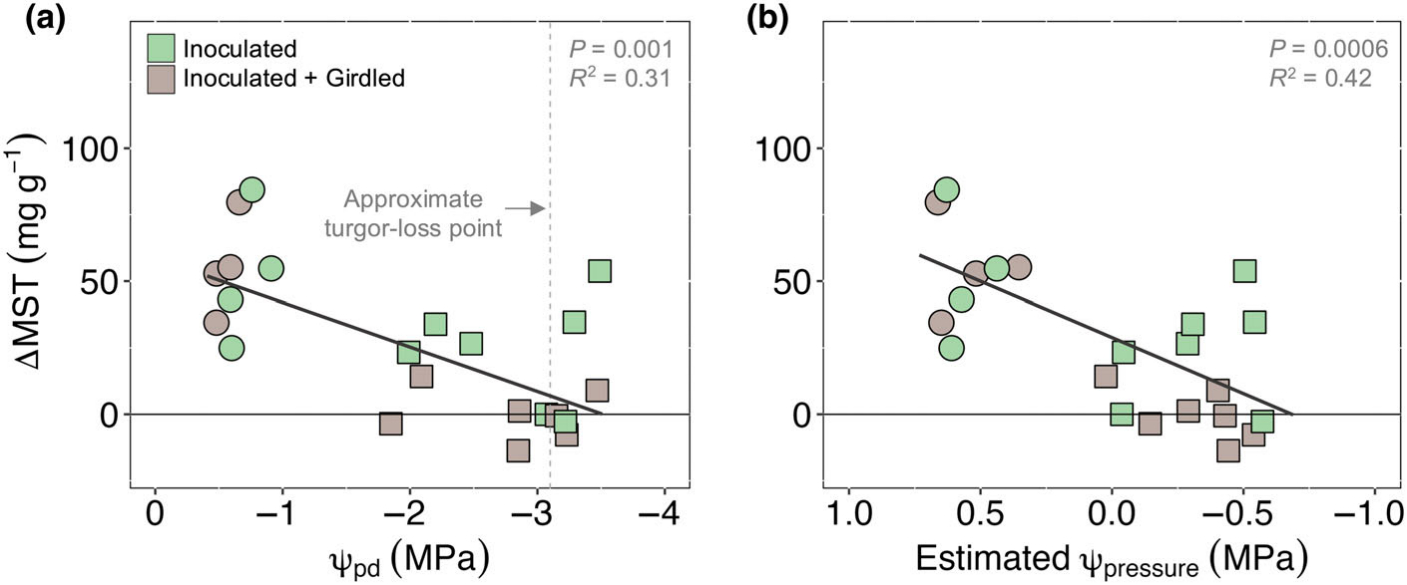
Mono‐ and sesquiterpene (MST) induction becomes constrained with decreasing water potential (a) and pressure potential (b) in *Pinus edulis*. Relationship of inner bark MST induction with shoot predawn water potential (a; ψ_pd_, MPa) and estimated inner bark pressure potential (b; Estimated ψ_pressure_, MPa) for inoculated (light green) and inoculated + girdled (light brown) trees. Trees were either watered (circles) or drought‐stressed (squares). Change in MST concentration (ΔMST, mg g^−1^) is the difference between post‐ and preinoculation. The vertical dashed gray line in (a) marks the approximate ψ_pd_ associated with the turgor‐loss point for *P. edulis* (−3.1 MPa, Meinzer *et al*., 2014).

Trees induced less MST as they experienced more negative ψ_pd_ and as they neared the turgor‐loss point for *P. edulis* (−3.1 MPa; Meinzer *et al*., 2014; Fig. 7a). Estimated inner bark ψ_pressure_ explained 42% of the variation in MST induction (Fig. 7b; *P* = 0.0006). When estimated ψ_pressure_ was greater than zero, trees were able to induce MST concentrations four times greater than when estimated ψ_pressure_ was negative (49.3 ± 7.2 and 11.9 ± 6.0 mg g^−1^, respectively). It is worth noting that for the trees most severely water‐stressed (ψ_pressure_ ≤ −0.4 MPa) and girdled (*n* = 4), the induced MST response was consistently low and near‐zero, but the absence of girdling (*n* = 3) led to significant variability in the terpene response (Fig. 7b). This notable variance within inoculated trees (I) suggests that for some trees, connectivity between the inoculation site and other organs of the tree may have buffered the effects of ψ_pressure_ alone, though it is unclear what mechanisms supported such high induction for two of these nongirdled trees.

## Discussion

Here, we provide multiple lines of evidence that drought exerts an appreciable constraint on the induced MST response of *P. edulis* to simulated bark beetle attack (Fig. 3a,b), but not through carbon substrate limitations *per se*. Because preformed resin is the dominant source of the induced MST response in *P. edulis* (Fig. 6a), drought‐constraints on defense arose through effects on the stored terpene pool, specifically by hindering effective resin transport (Fig. 7a,b). Taken together at the timescales studied here, drought‐induced constraints on MST defense in *P. edulis* appear largely biophysical through water limitation rather than metabolic and limited by carbon reserves (Fig. 1).

Current tree mortality frameworks suggest that defensive failure may be driven by drought‐induced reductions in NSC availability – either from depletion of locally stored carbon reserves and/or hindered transport of distal reserves (Sevanto, 2014; Salmon *et al*., 2018; Huang *et al*., 2020a; McDowell *et al*., 2022). In contrast with H1, however, our empirical data suggest that for species adapted to xeric environments, like *P. edulis*, NSC source (local or distal) appears relatively unimportant because NSC reserves matter less for an induced defense than is commonly assumed. Our stoichiometric estimates of maximum potential *de novo* synthesis reveal that even if all NSC available at the time of inoculation were used for MST synthesis, only 15% of the induction observed could be accounted for in unstressed trees (Fig. 6a). This is in stark contrast with H2 (that the induced MST response of *P. edulis* is predominately of *de novo* origin), and points toward the importance of existing resin stores as a major contributor to – and determinant of – the overall induced response. If across *Pinus*, induced terpene defenses originate predominantly from constitutively made resins stored long‐term, this may help explain correlations between constitutive and induce defenses for several *Pinus* species (Boone *et al*., 2011; Howe *et al*., 2020). Further, it may help explain why terpenes are less validated by the growth‐differentiation balance hypothesis (a substrate‐based model; Herms & Mattson, 1992) compared to other defense classes (Koricheva *et al*., 1998) as terpenes may be largely uncoupled from instantaneous substrate availability in terpene storing species like *Pinus*. Though we were unable to quantify diterpenes (a major component of resin in several *Pinus* species; Keefover‐Ring *et al*., 2016; Mason *et al*., 2017) in the present study, inclusion of diterpenes in our stoichiometric calculations would further reduce the proportion of *de novo* MST that could be supported by local NSC. This is because in *P. edulis*, fungal inoculation induces diterpenes with similar percent increases as monoterpenes and sesquiterpenes, and diterpenes comprise > 50 % of the resin (S. C. Malone & A. M. Trowbridge, unpublished). Therefore, the stoichiometric calculations presented here likely overestimate the proportion of MST that can be made *de novo*, further emphasizing the paramount role of stored resin toward the induced response.

Our data show that trees induced lower MST concentrations when they possessed more negative shoot water potentials (Figs 3a, 7a) and lower pressure potentials in the inner bark (Fig. 7b) (H3). Prior work suggests that as tree water pools decrease, turgor within epithelial cells may also decrease, increasing the intercellular space within resin ducts and reducing the pressure exerted on resin inside (Vité, 1961). Xylem tracheids may also undergo slight shrinkage (Neher, 1993), further compounding the negative effects of lower water pools on resin pressure. Thus, we conclude that watered trees exerted greater pressure on stem resin stores (Lorio *et al*., 1995; Rissanen *et al*., 2016, 2021) and this in turn allowed for a more effective transport of resin terpenes to the site of inoculation. While it is possible that NSC stored in xylem parenchyma provided additional substrate for *de novo* terpene synthesis, whether this alternative NSC source can resolve observed terpene differences among drought‐stressed and watered trees and whether they can explain the 85% of terpene induction unaccounted for in watered trees remains unlikely. To test whether xylem NSC could support defense synthesis in the present study, we quantified xylem NSC from a subset of girdled trees and found that xylem NSC did not change in response to inoculation, drought, or their interaction, nor were xylem NSC correlated with MST induction (data not shown). Therefore, in contrast to processes like regrowth and respiration (Carbon *et al*., 2013; Peltier *et al*., 2023), our preliminary data suggest that xylem NSC reserves are not mobilized to support defense synthesis within the phloem. Taken together, we conclude that the MST response of *P. edulis* to fungal challenge was chiefly modulated by water status while carbon reserves may only play indirect roles in so much as they can alter cell turgor (Sapes *et al*., 2021), influence root water uptake (Galvez *et al*., 2011; Wiley *et al*., 2019; Blumstein *et al*., 2023), and/or influence resin exudation (Rissanen *et al*., 2019). Additionally, the weak response of MST induction to girdling suggest that induction is localized (Mason *et al*., 2017) and largely independent of distal processes related to reserve allocation in response to biotic attack (Goodsman *et al*., 2013; Wiley *et al*., 2016).

That *P. edulis* rely substantially on preformed defenses for the induced response may not be surprising when considering the arid environment in which *P. edulis* have evolved. While prior work has assessed the influence of climate and resource availability on biotically induced terpene concentrations across conifers species (Howe *et al*., 2020), the effect of environment on induction strategy (i.e. contributions of *de novo* vs stored resin) has yet to be considered but may offer useful insight into the various mechanisms constraining *Pinus* defenses during drought. Defensive strategies favoring *de novo* induction in response to herbivory (rather than ongoing constitutive synthesis) may be less costly over the lifespan of a tree (Karban, 2011), but for trees occurring in stressful environments, immediate access to carbon substrate for defense synthesis upon herbivory is not guaranteed. Recent evidence suggests that species growing in water‐limited environments might not possess significant carbon reserve ‘buffers’ (Blumstein *et al*., 2023) for use during extended dry periods when carbon assimilation is limited. To mitigate this limitation, it follows that arid‐adapted *Pinus* species may rely more heavily on preformed stored resin toward an induced response and, therefore, may produce and store greater amounts of preformed resin defenses when conditions allow. By contrast, *Pinus* species adapted to mesic environments may rely more heavily on *de novo* synthesis for the induced response where terpene induction and NSC reserves may be more tightly coupled (e.g. *P. contorta*; Goodsman *et al*., 2013). Parametrizing contributions of *de novo* terpenes to the induced response within and across genera may help conceptualize how to model conifer defense processes under drought.

Our stoichiometric calculations estimate the maximum concentration of MST that can be produced *de novo*, but we suspect that the actual contribution of *de novo* synthesis to the overall induced MST response in *P. edulis* is even less. Both watered and drought‐stressed trees used 82% of preinoculation NSC, yet drought‐stressed trees exhibited minimal induction suggesting that the NSC used was not entirely for MST synthesis. If not for MST synthesis, then how was this NSC used? One possible explanation is that drought forced tradeoffs where trees invested NSC in metabolic processes related to maintenance respiration and drought resistance (i.e. hydraulic recovery, etc.) rather than defense (Fox *et al*., 2018; Klein *et al*., 2018; Tomasella *et al*., 2020). Another possible explanation is that in *P. edulis*, much of the NSC consumed in response to a biotic challenge are used for aspects of defense other than MST synthesis (i.e. synthesis of diterpenes, phenylpropanoids, lignin, degradation enzymes, and/or phytohormones, etc.) (Franceschi *et al*., 2005; Rigsby *et al*., 2019; Hundacker *et al*., 2024). Future work should explore how and where these carbon reserves are being used.

### Conclusion

Our data explicitly show that drought constrains the induced MST defense response within the inner bark of *P. edulis* not through limitations on carbon substrate availability, but through water effects on turgor and resin transport. Though the induced response represents a strong carbon sink, the NSC concentrations consumed accounted for only 15% of the MST induction observed at maximum, and probably even less. We conclude the induced response in *P. edulis* is primarily of stored origin and that drought‐constraints on induction are largely biophysical, likely a consequence of low duct pressure due to diminishing water reserves within the stem. *Pinus edulis* that induce and exude more resin are more likely to survive attack by *I. confusus* bark beetles, suggesting that under certain situations, tree susceptibility may be directly linked to water limitation, and not carbon limitation as is often assumed (Huang *et al*., 2020a; McDowell *et al*., 2022). Our work offers a blueprint for how to explicitly consider and account for the relative contribution of *de novo* vs stored resin in the induced defense response. Accounting for these individual traits and their unique responses to drought in conifers across diverse systems will be critical for improving our predictability of massive tree mortality events in the face of a rapidly changing environment.

## Competing interests

None declared.

## Author contributions

SCM and AMT designed the experiment with input from KAM, DLS and HDA; SCM collected the data; SCM conducted terpene analysis and PSC and CRO conducted NSC analysis; SCM analyzed the data with input from AMT, SML, RAT, HDA and KAM; SCM wrote the first draft of the manuscript; all authors contributed significantly to revisions and the final manuscript.

## Supporting information


**Fig. S1** Tree transplant information.
**Fig. S2** Net photosynthesis and stomatal conductance in response to predawn water potential.
**Fig. S3** Girdle and inoculation treatment information.
**Fig. S4** Mono‐ and sesquiterpene concentrations in response to girdle and inoculation treatments for watered and drought‐stressed trees.
**Fig. S5** Relative mono‐ and sesquiterpene loss due to girdling alone in response to predawn water potential.
**Fig. S6** Drought depleted inner bark starch concentrations but did not affect sugar concentrations.
**Fig. S7** Girdle and inoculation treatment effects on glucose, fructose, and sucrose.
**Fig. S8** Total nonstructural carbohydrates available preinoculation were not correlated with induced mono‐ and sesquiterpene concentrations.
**Methods S1** Net photosynthesis and stomatal conductance measurements.
**Methods S2** Quantification of mono‐ and sesquiterpenes and nonstructural carbohydrates.
**Methods S3** Determining the glucose cost required to synthesize 1 g mono‐ and sesquiterpene.
**Methods S4** Estimates of maximum potential mono‐ and sesquiterpene induction assuming 100% availability of preinoculation nonstructural carbohydrate concentrations.
**Table S1** Individual mono‐ and sesquiterpene concentrations in response to drought, fungal inoculation, and their interaction.
**Table S2** ANOVA analysis of nonstructural carbohydrate dynamics on mono‐ and sesquiterpene induction.
Please note: Wiley is not responsible for the content or functionality of any Supporting Information supplied by the authors. Any queries (other than missing material) should be directed to the *New Phytologist* Central Office.

## Data Availability

The datasets generated and analyzed during the current study are available in the Dryad repository doi: 10.5061/dryad.tmpg4f57n.

## References

[nph20218-bib-0001] Adams HD , Germino MJ , Breshears DD , Barron‐Gafford GA , Guardiola‐Claramonte M , Zou CB , Huxman TE . 2013. Nonstructural leaf carbohydrate dynamics of *Pinus edulis* during drought‐induced tree mortality reveal role for carbon metabolism in mortality mechanism. New Phytologist 197: 1142–1151. 23311898 10.1111/nph.12102

[nph20218-bib-0002] Anderegg WRL , Hicke JA , Fisher RA , Allen CD , Aukema J , Bentz B , Hood S , Lichstein JW , Macalady AK , McDowell N *et al*. 2015. Tree mortality from drought, insects, and their interactions in a changing climate. New Phytologist 208: 674–683. 26058406 10.1111/nph.13477

[nph20218-bib-0003] Anderson MJ . 2017. Permutational multivariate analysis of variance (permanova). In: Balakrishnan N , Everitt B , Piegorsch W , Ruggeri F , Teugels JL , eds. Wiley StatsRef: statistics reference online. New York, NY, USA: John Wiley & Sons Ltd, 1–15.

[nph20218-bib-0004] Banerjee A , Sharkey TD . 2014. Methylerythritol 4‐phosphate (MEP) pathway metabolic regulation. Natural Product Reports 31: 1043–1055. 24921065 10.1039/c3np70124g

[nph20218-bib-0080] Bartlett MK , Scoffoni C , Sack L . 2012. The determinants of leaf turgor loss point and prediction of drought tolerance of species and biomes: a global meta‐analysis. Ecology Letters 15: 393–405. 22435987 10.1111/j.1461-0248.2012.01751.x

[nph20218-bib-0005] Blumstein M , Gersony J , Martínez‐Vilalta J , Sala A . 2023. Global variation in nonstructural carbohydrate stores in response to climate. Global Change Biology 29: 1854–1869. 36583374 10.1111/gcb.16573

[nph20218-bib-0006] Boone CK , Aukema BH , Bohlmann J , Carroll AL , Raffa KF . 2011. Efficacy of tree defense physiology varies with bark beetle population density: a basis for positive feedback in eruptive species. Canadian Journal of Forest Research 41: 1174–1188.

[nph20218-bib-0007] Breshears DD , Carroll CJW , Redmond MD , Wion AP , Allen CD , Cobb NS , Meneses N , Field JP , Wilson LA , Law DJ *et al*. 2018. A dirty dozen ways to die: metrics and modifiers of mortality driven by drought and warming for a tree species. Frontiers in Forests and Global Change 1: 4.

[nph20218-bib-0008] Breshears DD , Cobb NS , Rich PM , Price KP , Allen CD , Balice RG , Romme WH , Kastens JH , Floyd ML , Belnap J *et al*. 2005. Regional vegetation die‐off in response to global‐change‐type drought. Proceedings of the National Academy of Sciences, USA 102: 15144–15148. 10.1073/pnas.0505734102 PMC1250231 16217022

[nph20218-bib-0009] Byun‐McKay A , Godard KA , Toudefallah M , Martin DM , Alfaro R , King J , Bohlmann J , Plant AL . 2006. Wound‐induced terpene synthase gene expression in Sitka spruce that exhibit resistance or susceptibility to attack by the white pine weevil. Plant Physiology 140: 1009–1021. 16415217 10.1104/pp.105.071803 PMC1400563

[nph20218-bib-0010] Carbon MS , Czimczik CI , Keenan TF , Murakami PF , Pederson N , Schaberg PG , Xu X , Richardson AD . 2013. Age, allocation and availability of nonstructural carbon in mature red maples. New Phytologist 200: 1145–1155. 24032647 10.1111/nph.12448

[nph20218-bib-0011] Chen R , He X , Chen J , Gu T , Liu P , Xu T , Teale SA , Hao D . 2019. Traumatic resin duct development, terpenoid formation, and related synthase gene expression in *Pinus massoniana* under feeding pressure of *Monochamus alternatus* . Journal of Plant Growth Regulation 38: 897–908.

[nph20218-bib-0012] Coley PD , Bryant JP , Chapin FS . 1985. Resource availability and plant antiherbivore defense. Science 230: 895–899. 17739203 10.1126/science.230.4728.895

[nph20218-bib-0013] De Vries FP . 1974. Products, requirements and efficiency of biosynthesis a quantitative approach. Journal of Theoretical Biology 45: 339–377. 4367755 10.1016/0022-5193(74)90119-2

[nph20218-bib-0014] Dickman LT , McDowell NG , Sevanto S , Pangle RE , Pockman WT . 2015. Carbohydrate dynamics and mortality in a piñon‐juniper woodland under three future precipitation scenarios. Plant, Cell & Environment 38: 729–739. 10.1111/pce.12441 25159277

[nph20218-bib-0015] Erbilgin N , Zanganeh L , Klutsch JG , Chen S‐H , Zhao S , Ishangulyyeva G , Burr SJ , Gaylord M , Hofstetter R , Keefover‐Ring K *et al*. 2021. Combined drought and bark beetle attacks deplete non‐structural carbohydrates and promote death of mature pine trees. Plant, Cell & Environment 44: 3636–3651. 10.1111/pce.14197 34612515

[nph20218-bib-0016] Floyd ML , Clifford M , Cobb NS , Hannah D , Delph R , Ford P , Turner D . 2009. Relationship of stand characteristics to drought‐induced mortality in three southwestern piñon‐juniper woodlands. Ecological Applications 5: 1223–1230. 10.1890/08-1265.1 19688929

[nph20218-bib-0017] Fox H , Doron‐Faigenboim A , Kelly G , Bourstein R , Attia Z , Zhou J , Moshe Y , Moshelion M , David‐Schwartz R . 2018. Transcriptome analysis of *Pinus halepensis* under drought stress and during recovery. Tree Physiology 38: 423–441. 29177514 10.1093/treephys/tpx137 PMC5982726

[nph20218-bib-0018] Franceschi VR , Krokene P , Christiansen E , Krekling T . 2005. Anatomical and chemical defenses of conifer bark against bark beetles and other pests. New Phytologist 167: 353–376. 15998390 10.1111/j.1469-8137.2005.01436.x

[nph20218-bib-0019] Galvez DA , Landhäusser SM , Tyree MT . 2011. Root carbon reserve dynamics in aspen seedlings: does simulated drought induce reserve limitation? Tree Physiology 31: 250–257. 21444372 10.1093/treephys/tpr012

[nph20218-bib-0020] Gaylord ML , Kolb TE , Pockman WT , Plaut JA , Yepez EA , Macalady AK , Pangle RE , McDowell NG . 2013. Drought predisposes piñon‐juniper woodlands to insect attacks and mortality. New Phytologist 198: 567–578. 23421561 10.1111/nph.12174

[nph20218-bib-0021] Gershenzon J , Murtagh GJ , Croteau R . 1993. Absence of rapid terpene turnover in several diverse species of terpene‐accumulating plants. Oecologia 96: 583–592. 28312466 10.1007/BF00320517

[nph20218-bib-0022] Gersony J , Holbrook M . 2022. Phloem turgor is maintained during severe drought in *Ricinus communis* . New Phytologist 45: 2898–2905. 10.1111/pce.14401 35854434

[nph20218-bib-0023] Goodsman DW , Lusebrink I , Landhäusser SM , Erbilgin N , Lieffers VJ . 2013. Variation in carbon availability, defense chemistry and susceptibility to fungal invasion along the stems of mature trees. New Phytologist 197: 586–594. 23157572 10.1111/nph.12019

[nph20218-bib-0081] Guérard N , Maillard P , Bréchet C , Lieutier F , Dreyer E . 2007. Do trees use reserve or newly assimilated carbon for their defense reactions? A ^13^C labeling approach with young Scots pines inoculated with a bark‐beetle‐associated fungus (*Ophiostoma brunneo ciliatum*) Les arbres utilisentils du carbone de réserve ou du c. Annals of Forest Science 64: 601–608.

[nph20218-bib-0024] Hammond WM , Yu K , Wilson LA , Will RE , Anderegg WRL , Adams HD . 2019. Dead or dying? Quantifying the point of no return from hydraulic failure in drought‐induced tree mortality. New Phytologist 223: 1834–1843. 31087656 10.1111/nph.15922 PMC6771894

[nph20218-bib-0025] Herms DA , Mattson WJ . 1992. The dilemma of plants: to grown or defend. The Quarterly Review of Biology 67: 283–336.

[nph20218-bib-0026] Howe M , Mason CJ , Gratton C , Keefover‐Ring K , Wallin K , Yanchuk A , Zhu J , Raffa KF . 2020. Relationships between conifer constitutive and inducible defenses against bark beetles change across levels of biological and ecological scale. Oikos 129: 1093–1107.

[nph20218-bib-0027] Howe M , Yanchuk A , Wallin KF , Raffa KF . 2024. Quantification of heritable variation in multiple lodgepole pine chemical and physical traits that contribute to defense against mountain pine beetle (*Dendroctonus ponderosae*). Forest Ecology and Management 553: 121660.

[nph20218-bib-0028] Huang J , Hartmann H , Ogaya R , Schöning I , Reichelt M , Gershenzon J , Peñuelas J . 2023. Hormone and carbohydrate regulation of defense secondary metabolites in a Mediterranean forest during drought. Environmental and Experimental Botany 209: 105298.

[nph20218-bib-0029] Huang J , Kautz M , Trowbridge AM , Hammerbacher A , Raffa KF , Adams HD , Goodsman DW , Xu C , Meddens AJH , Kandasamy D *et al*. 2020a. Tree defence and bark beetles in a drying world: carbon partitioning, functioning and modelling. New Phytologist 225: 26–36. 31494935 10.1111/nph.16173

[nph20218-bib-0030] Huang J , Rücker A , Schmidt A , Gleixner G , Gershenzon J , Trumbore S , Hartmann H . 2020b. Production of constitutive and induced secondary metabolites is coordinated with growth and storage in Norway spruce saplings. Tree Physiology 40: 928–942. 32268379 10.1093/treephys/tpaa040 PMC7325531

[nph20218-bib-0031] Hudgins JW , Christiansen E , Franceschi VR . 2004. Induction of anatomically based defense responses in stems of diverse conifers by methyl jasmonate: a phylogenetic perspective. Tree Physiology 24: 251–264. 14704135 10.1093/treephys/24.3.251

[nph20218-bib-0032] Hundacker J , Linda T , Hilker M , Lortzing V , Bittner N . 2024. The impact of insect egg deposition on *Pinus sylvestris* transcriptomic and phytohormonal responses to larval herbivory. Tree Physiology 44: tpae008. 38227779 10.1093/treephys/tpae008 PMC10878248

[nph20218-bib-0033] Jactel H , Petit J , Desprez‐Loustau ML , Delzon S , Piou D , Battisti A , Koricheva J . 2012. Drought effects on damage by forest insects and pathogens: a meta‐analysis. Global Change Biology 18: 267–276.

[nph20218-bib-0034] Karban R . 2011. The ecology and evolution of induced resistance against herbivores. Functional Ecology 25: 339–347.

[nph20218-bib-0035] Keefover‐Ring K , Trowbridge AM , Mason CJ , Raffa KF . 2016. Rapid induction of multiple terpenoid groups by ponderosa pine in response to bark beetle‐associated fungi. Journal of Chemical Ecology 42: 1–12. 26662358 10.1007/s10886-015-0659-6

[nph20218-bib-0036] Klein T , Zeppel MJB , Anderegg WRL , Bloemen J , De Kauwe MG , Hudson P , Ruehr NK , Powell TL , von Arx G , Nardini A . 2018. Xylem embolism refilling and resilience against drought‐induced mortality in woody plants: processes and trade‐offs. Ecological Research 33: 839–855.

[nph20218-bib-0037] Koepke DF , Kolb TE . 2013. Cavitation at a forest‐woodland ecotone. Forest Science 59: 524–535.

[nph20218-bib-0038] Kolb TE , Fettig CJ , Ayres MP , Bentz BJ , Hicke JA , Mathiasen R , Stewart JE , Weed AS . 2016. Observed and anticipated impacts of drought on forest insects and diseases in the United States. Forest Ecology and Management 380: 321–334.

[nph20218-bib-0039] Kopper BJ , Illman BL , Kersten PJ , Klepzig KD , Raffa KF . 2005. Effects of diterpene acids on components of a conifer bark beetle‐fungal interaction: tolerance by *Ips pini* and sensitivity by its associate *Ophiostoma ips* . Environmental Entomology 34: 486–493.

[nph20218-bib-0040] Krokene P . 2015. Conifer defense and resistance to bark beetles. In: Vega FE , Hofstetter RW , eds. Bark beetles. San Diego, CA, USA: Academic Press, 177–207.

[nph20218-bib-0082] Koricheva J , Larsson S , Haukioja E , Keinänen M . 1998. Regulation of woody plant secondary metabolism by resource availability: hypothesis testing by means of meta‐analysis. Oikos 160: 212–226.

[nph20218-bib-0041] Krokene P , Nagy NE . 2012. Anatomical aspects of resin‐based defences in pine. In: Fett‐Neto A , Rodrigues‐Corrêa K , eds. Pine resin: biology, chemistry and applications. Kerala, India: Research Signpost, 67–86.

[nph20218-bib-0042] Landhäusser SM , Chow PS , Dickman LT , Furze ME , Kuhlman I , Schmid S , Wiesenbauer J , Wild B , Gleixner G , Hartmann H *et al*. 2018. Standardized protocols and procedures can precisely and accurately quantify non‐structural carbohydrates. Tree Physiology 38: 1764–1778. 30376128 10.1093/treephys/tpy118 PMC6301340

[nph20218-bib-0043] Lenth RV . 2022. emmeans: estimated marginal means, aka least‐squares means . R package v.1.10.1. [WWW document] URL https://CRAN.R-project.org/package=emmeans [accessed 24 January 2024].

[nph20218-bib-0044] Lewinsohn E , Gijzen M , Croteau R . 1991. Defense mechanisms of conifers. Plant Physiology 96: 44–49. 16668184 10.1104/pp.96.1.44 PMC1080711

[nph20218-bib-0045] Lorio PL , Stephen FM , Paine TD . 1995. Environment and ontogeny modify loblolly pine respone to induced acute water deficits and bark beetle attack. Forest Ecology and Management 73: 97–110.

[nph20218-bib-0046] Martin D , Tholl D , Gershenzon J , Bohlmann J . 2002. Methyl jasmonate induces traumatic resin ducts, terpenoid resin biosynthesis, and terpenoid accumulation in developing xylem of Norway spruce stems. Plant Physiology 129: 1003–1018. 12114556 10.1104/pp.011001 PMC166496

[nph20218-bib-0047] Mason CJ , Villari C , Keefover‐Ring K , Jagemann S , Zhu J , Bonello P , Raffa KF . 2017. Spatial and temporal components of induced plant responses in the context of herbivore life history and impact on host. Functional Ecology 31: 2034–2050.

[nph20218-bib-0048] McDowell NG , Beerling DJ , Breshears DD , Fisher RA , Raffa KF , Stitt M . 2011. The interdependence of mechanisms underlying climate‐driven vegetation mortality. Trends in Ecology & Evolution 26: 523–532. 21802765 10.1016/j.tree.2011.06.003

[nph20218-bib-0049] McDowell NG , Sapes G , Pivovaroff A , Adams HD , Allen CD , Anderegg WRL , Arend M , Breshears DD , Brodribb T , Choat B *et al*. 2022. Mechanisms of woody‐plant mortality under rising drought, CO_2_ and vapour pressure deficit. Nature Reviews Earth & Environment 3: 294–308.

[nph20218-bib-0050] Meinzer FC , Woodruff DR , Marias DE , McCulloh KA , Sevanto S . 2014. Dynamics of leaf water relations components in co‐occurring iso‐ and anisohydric conifer species. Plant, Cell & Environment 37: 2577–2586. 10.1111/pce.12327 24661116

[nph20218-bib-0051] Miller RH , Berryman AA . 1986. Carbohydrate allocation and mountain pine beetle attack in girdled lodgepole pine. Canadian Journal of Forest Research 16: 1036–1040.

[nph20218-bib-0078] Münch E . 1930. Stoffbewegungen in der Pflanze. Jena, Germany: Gustav Fischer.

[nph20218-bib-0052] Negrón J , Wilson J . 2003. Attributes associated with probability of infestation by the piñon ips, *Ips confusus* (Coleoptera: Scolytidae), in piñon pine, *Pinus edulis* . Western North American Naturalist 4: 440–451.

[nph20218-bib-0053] Neher HV . 1993. Effects of pressures inside Monterey pine trees. Trees 8: 9–17.

[nph20218-bib-0054] Oksanen J , Guillaume F , Friendly M , Kindt R , Legendre P , GcGlinn D , Minchin PR , O'Hara RB , Simpson L , Solymos P *et al*. 2020. vegan: community ecology package . R package v.2.6‐4. [WWW document] URL https://CRAN.R-project.org/package=vegan [accessed 24 January 2024].

[nph20218-bib-0055] Peltier DM , Carbone MS , McIntire CD , Robertson N , Thompson RA , Malone S , LeMoine J , Richardson AD , McDowell NG , Adams HD *et al*. 2023. Carbon starvation following a decade of experimental drought consumes old reserves in *Pinus edulis* . New Phytologist 240: 92–104. 37430467 10.1111/nph.19119

[nph20218-bib-0056] Raffa KF , Aukema BH , Erbilgin N , Klepzig KD , Wallin KF . 2005. Interactions among conifer terpenoids and bark beetles across multiple levels of scale: an attempt to understand links between population patterns and physiological processes. Recent Advances in Phytochemistry 39: 79–118.

[nph20218-bib-0057] Raffa KF , Grégoire JC , Lindgren BS . 2015. Natural history and ecology of bark beetles. In: Vega FE , Hofstetter RW , eds. Bark beetles. San Diego, CA, USA: Academic Press, 1–40.

[nph20218-bib-0058] Rigsby CM , Shoemaker EE , Mallinger MM , Orians CM , Preisser EL . 2019. Conifer responses to a stylet‐feeding invasive herbivore and induction with methyl jasmonate: impact on the expression of induced defences and a native folivore. Agricultural and Forest Entomology 21: 227–234.

[nph20218-bib-0059] Rissanen K , Hölttä T , Bäck J , Rigling A , Wermelinger B , Gessler A . 2021. Drought effects on carbon allocation to resin defences and on resin dynamics in old‐grown scots pine. Environmental and Experimental Botany 185: 104410.

[nph20218-bib-0060] Rissanen K , Hölttä T , Barreira LFM , Hyttinen N , Kurtén T , Bäck J . 2019. Temporal and spatial variation in scots pine resin pressure and composition. Frontiers in Forests and Global Change 2: 1–14.

[nph20218-bib-0061] Rissanen K , Hölttä T , Vanhatalo A , Aalto J , Nikinmaa E , Rita H , Bäck J . 2016. Diurnal patterns in Scots pine stem oleoresin pressure in a boreal forest. Plant, Cell & Environment 39: 527–538. 10.1111/pce.12637 26385487

[nph20218-bib-0062] Roth M , Hussain A , Cale JA , Erbilgin N . 2018. Successful colonization of lodgepole pine trees by mountain pine beetle increased monoterpene production and exhausted carbohydrate reserves. Journal of Chemical Ecology 44: 209–214. 29302834 10.1007/s10886-017-0922-0

[nph20218-bib-0063] Sala A , Piper F , Hoch G . 2010. Physiological mechanisms of drought‐induced tree mortality are far from being resolved. New Phytologist 186: 274–281. 20409184 10.1111/j.1469-8137.2009.03167.x

[nph20218-bib-0064] Salmon Y , Dietrich L , Sevanto S , Hölttä T , Dannoura M , Epron D . 2018. Drought impacts on tree phloem: from cell‐level responses to ecological significance. Tree Physiology 39: 173–191. 10.1093/treephys/tpy153 30726983

[nph20218-bib-0065] Sapes G , Demaree P , Lekberg Y , Sala A . 2021. Plant carbohydrate depletion impairs water relations and spreads via ectomycorrhizal networks. New Phytologist 229: 3172–3183. 33280134 10.1111/nph.17134

[nph20218-bib-0066] Sevanto S . 2014. Phloem transport and drought. Journal of Experimental Botany 65: 1751–1759. 24431155 10.1093/jxb/ert467

[nph20218-bib-0067] Sevanto S , McDowell NG , Dickman LT , Pangle R , Pockman WT . 2014. How do trees die? A test of the hydraulic failure and carbon starvation hypotheses. Plant, Cell & Environment 37: 153–161. 10.1111/pce.12141 PMC4280888 23730972

[nph20218-bib-0068] Thompson RA , Adams HD , Breshears DD , Collins AD , Dickman LT , Grossiord C , Manrique‐Alba À , Peltier DM , Ryan MG , Trowbridge AM *et al*. 2023. No carbon storage in growth‐limited trees in a semi‐arid woodland. Nature Communications 14: 1959. 10.1038/s41467-023-37577-8 PMC10081995 37029120

[nph20218-bib-0069] Thompson RA , Malone SC , Peltier DMP , Six DL , Robertson N , de Oliveira CR , McIntire CD , Pockman WT , McDowell NG , Trowbridge AM *et al*. 2024. Local carbon reserves are insufficient for phloem terpene induction during drought in response to bark‐beetle associated fungi. New Phytologist 244: 654–669. 39149848 10.1111/nph.20051

[nph20218-bib-0070] Tomasella M , Petrussa E , Petruzzellis F , Nardini A , Casolo V . 2020. The possible role of non‐structural carbohydrates in the regulation of tree hydraulics. International Journal of Molecular Sciences 21: 144. 10.3390/ijms21010144 PMC6981889 31878253

[nph20218-bib-0071] Trapp S , Croteau R . 2001. Defensive resin biosynthesis in conifers. Annual Review of Plant Biology 52: 689–724. 10.1146/annurev.arplant.52.1.689 11337413

[nph20218-bib-0072] Trowbridge AM , Adams HD , Collins A , Dickman LT , Grossiord C , Hofland M , Malone S , Weaver DK , Sevanto S , Stoy PC *et al*. 2021. Hotter droughts alter resource allocation to chemical defenses in piñon pine. Oecologia 197: 921–938. 34657177 10.1007/s00442-021-05058-8 PMC8591002

[nph20218-bib-0073] Vité J . 1961. The influence of water supply on oleoresin exudation pressure and resistance to bark beetle attack in *Pinus ponderosa* . Boyce Thompson Institute 21: 31–66.

[nph20218-bib-0074] Wiley E , King CM , Landhäusser SM . 2019. Identifying the relevant carbohydrate storage pools available for remobilization in aspen roots. Tree Physiology 39: 1109–1120. 31094427 10.1093/treephys/tpz051

[nph20218-bib-0075] Wiley E , Rogers BJ , Hodgkinson R , Landhäusser SM . 2016. Nonstructural carbohydrate dynamics of lodgepole pine dying from mountain pine beetle attack. New Phytologist 209: 550–562. 26256444 10.1111/nph.13603

[nph20218-bib-0076] Wion AP , Breshears DD , Carroll CJW , Cobb NS , Hart SJ , Law DJ , Meneses N , Redmond MD . 2022. Dead again: predictions of repeat tree die‐off under hotter droughts confirm mortality thresholds for a dryland conifer. Environmental Research Letters 17: 074031.

[nph20218-bib-0077] Zulak KG , Bohlmann J . 2010. Terpenoid biosynthesis and specialized vascular cells of conifer defense. Journal of Integrative Plant Biology 52: 86–97. 20074143 10.1111/j.1744-7909.2010.00910.x

