# Supplementary material for: Water, not carbon, drives drought‐constraints on stem terpene defense against simulated bark beetle attack in Pinus edulis
Source: New Phytol. 2024 Oct 27;245(1):318–31. doi: 10.1111/nph.20218 (PMC11617656; doi:10.1111/nph.20218)
Supplement: Supplementary file 1 — Fig. S1 Tree transplant information. Fig. S2 Net photosynthesis and stomatal conductance in response to predawn water potential. Fig. S3 Girdle and inoculation treatment information. Fig. S4 Mono‐ and sesquiterpene concentrations in response to girdle and inoculation treatments for watered and drought‐stressed trees. Fig. S5 Relative mono‐ and sesquiterpene loss due to girdling alone in response to predawn water potential. Fig. S6 Drought depleted inner bark starch concentrations but did not affect sugar concentrations. Fig. S7 Girdle and inoculation treatment effects on glucose, fructose, and sucrose. Fig. S8 Total nonstructural carbohydrates available preinoculation were not correlated with induced mono‐ and sesquiterpene concentrations. Methods S1 Net photosynthesis and stomatal conductance measurements. Methods S2 Quantification of mono‐ and sesquiterpenes and nonstructural carbohydrates. Methods S3 Determining the glucose cost required to synthesize 1 g mono‐ and sesquiterpene. Methods S4 Estimates of maximum potential mono‐ and sesquiterpene induction assuming 100% availability of preinoculation nonstructural carbohydrate concentrations. Table S1 Individual mono‐ and sesquiterpene concentrations in response to drought, fungal inoculation, and their interaction. Table S2 ANOVA analysis of nonstructural carbohydrate dynamics on mono‐ and sesquiterpene induction. Please note: Wiley is not responsible for the content or functionality of any Supporting Information supplied by the authors. Any queries (other than missing material) should be directed to the New Phytologist Central Office. [file NPH-245-318-s001.docx]

## *New Phytologist* Supporting Information

Article title: Water, not carbon, drives drought-constraints on stem terpene defense against simulated bark beetle attack in *Pinus edulis*

Authors: Shealyn C. Malone, R. Alex Thompson, Pak S. Chow, Celso R. de Oliveira Jr., Simon M. Landhäusser, Diana L. Six, Katherine A. McCulloh, Henry D. Adams, Amy M. Trowbridge

Article acceptance date: 03 October 2024

The following Supporting Information is available for this article:

**Fig. S1** Tree transplant information.

**Fig. S2** Net photosynthesis and stomatal conductance in response to pre-dawn water potential.

**Fig. S3** Girdle and inoculation treatment information.

**Fig. S4** Mono- and sesquiterpene concentrations in response to girdle and inoculation treatments for watered and drought-stressed trees.

**Fig. S5** Relative mono- and sesquiterpene loss due to girdling alone in response to pre-dawn water potential.

**Fig. S6** Drought depleted inner bark starch concentrations but did not affect sugar concentrations.

**Fig. S7** Girdle and inoculation treatment effects on glucose, fructose, and sucrose.

**Fig. S8** Total non-structural carbohydrates available pre-inoculation were not correlated with induced mono- and sesquiterpene concentrations.

**Table S1** Individual mono- and sesquiterpene concentrations in response to drought, fungal inoculation, and their interaction

**Table S2** ANOVA analysis of non-structural carbohydrate dynamics on mono- and sesquiterpene induction

**Methods S1** Net photosynthesis and stomatal conductance measurements

**Methods S2** Quantification of mono- and sesquiterpenes and non-structural carbohydrates

**Methods S3** Determining the glucose cost required to synthesize 1 g mono- and sesquiterpene

**Methods S4** Estimates of maximum potential mono- and sesquiterpene induction assuming 100% availability of pre-inoculation non-structural carbohydrate concentrations

**Fig. S1** Mature, native-grown *Pinus edulis* were spade dug from southern Colorado during mid-October 2020 and subsequently balled-and-burlaped (a) for shipment to the University of Wisconsin-Madison (photo by Levi Heidrich). Upon arrival, trees were immediately transplanted into 70 L pots (b) with their native soil. On 4 March 2021, lower branches were removed from the bole (c) to create branchless sections to which girdle and inoculation treatments would be administered.

**
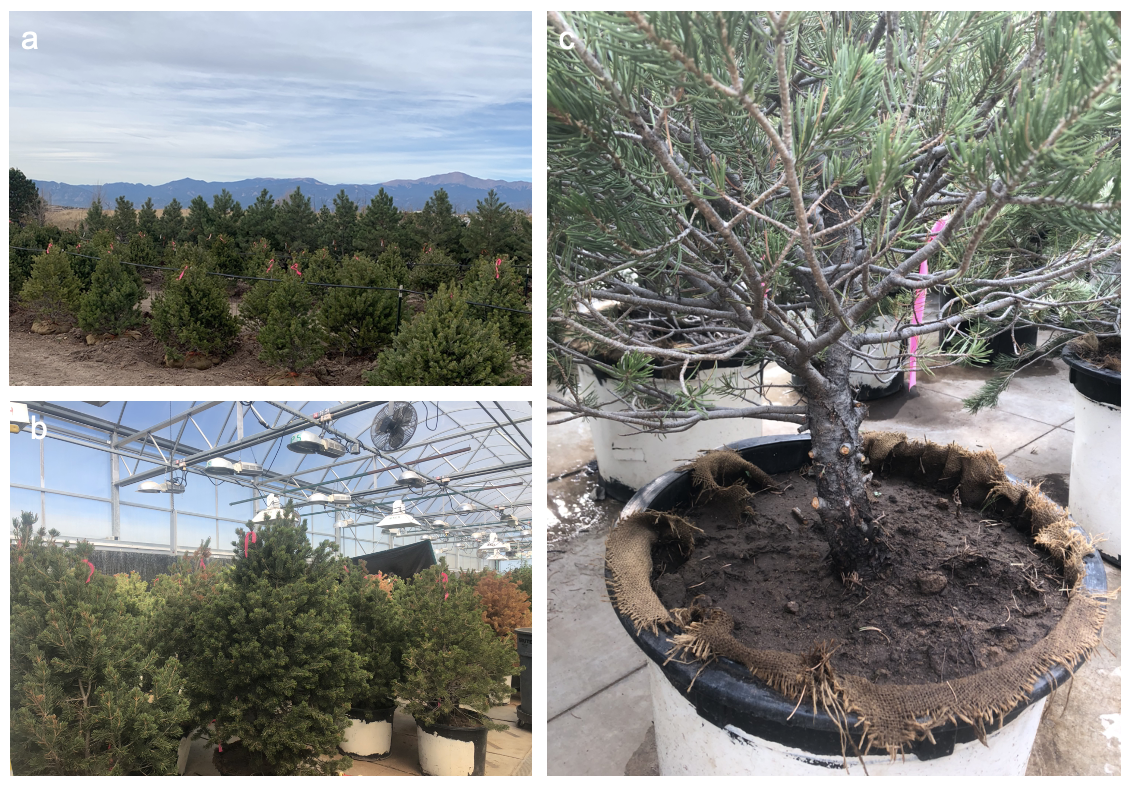
**

**Fig. S2** Net photosynthesis (a; *A*, μmol m^-2^ s^-1^) and stomatal conductance (b; *g_s_*, mol m^-2^ s^-1^) in response to pre-dawn water potential (ψ_pd_, MPa) in *Pinus edulis*. Measurements occurred mid-morning from current-year needles at three timepoints: pre-drought, mid-drought, and during the two-week inoculation window. Trees were assigned to one of two drought treatments: water (circles) or drought (squares). Note that all trees were watered pre-drought, independent of drought treatment.


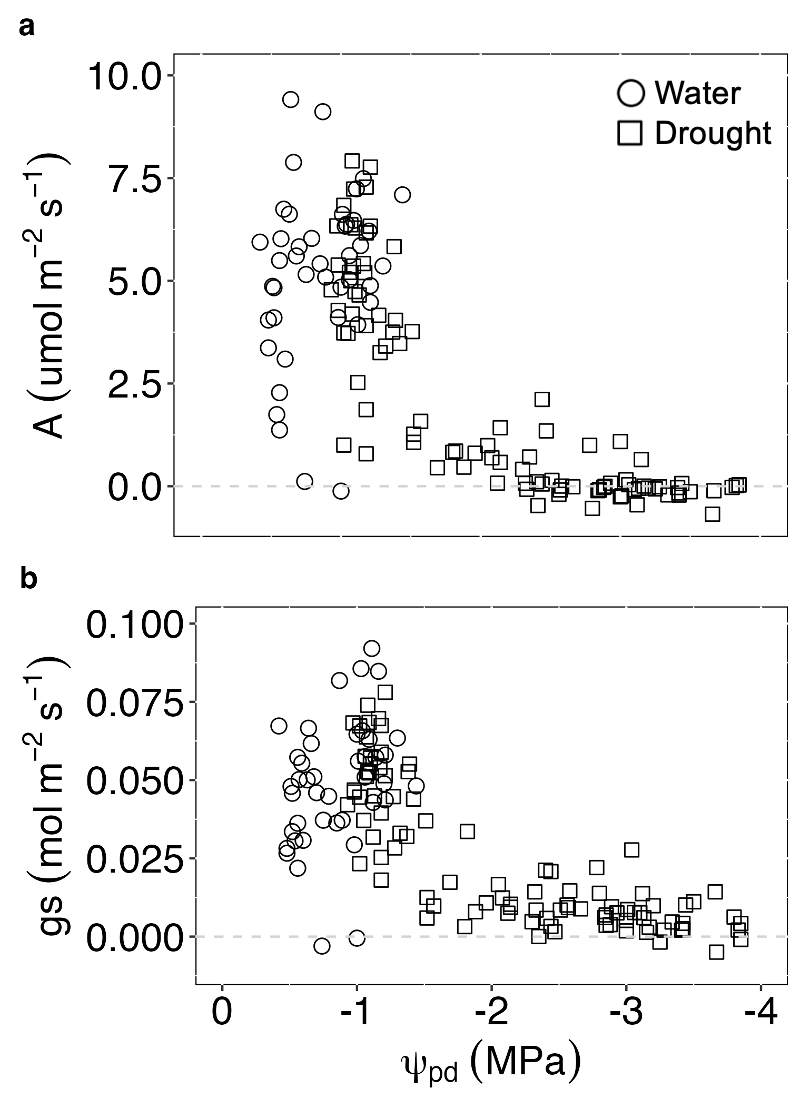


**Fig. S3** Girdle and inoculation treatments. The girdle treatment (a) was applied to a subset of *Pinus edulis* individuals. Girdles were applied along the mainstem around the ‘inoculation zone’ to impede bidirectional carbohydrate transport from distal sources (i.e*.*, crown and roots). The inoculation treatment was applied by removing two bark plugs, and to each, applying agar containing actively growing *Ophiostoma* sp. (b); for non-inoculated trees, the bark plugs were immediately reinserted without application of the fungal plug. Inner bark tissue was collected pre-drought and pre-inoculation (c) for determination of drought effects on mono- and sesquiterpenes (MST) and non-structural carbohydrates (NSC). Sampling occurred on separate sides of the stem. Inner bark tissue was sampled from the inoculation zone two weeks after the application of the inoculation treatment (d) for determination of girdle x inoculation effects on MST and NSC.


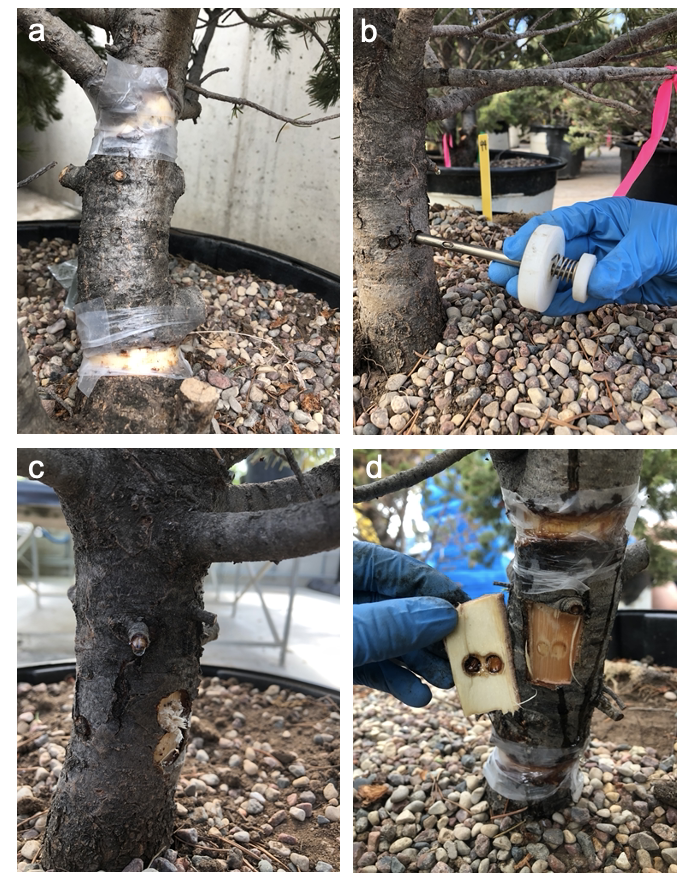


**Fig. S4** Mono- and sesquiterpene (MST) concentration in response to girdle and inoculation treatments for watered and drought-stressed *Pinus edulis*. Change in MST concentration ($\Delta$ MST, mg g^-1^) is the difference between post- and pre-inoculation. Error bars represent +/- SE and gray lowercase letters denote significant differences among group means, controlling for drought (*P* < 0.05, Tukey-HSD followed by ANOVA). Circles represent individual trees.


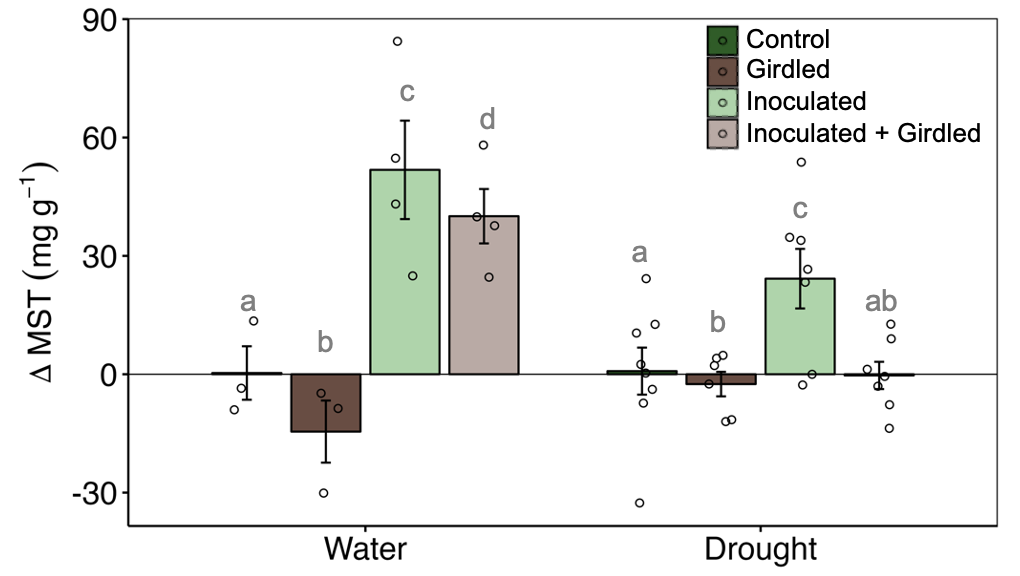


**Fig. S5** Relative mono- and sesquiterpene (MST) loss due to girdling in response to pre-dawn water potential (ψ_pd_, MPa) for *Pinus edulis* individuals in the girdle treatment (G). Relative MST loss was calculated by subtracting pre-inoculation MST concentration from post-inoculation concentration and dividing the difference by pre-inoculation concentration. This relationship was used to estimate the relative amount of girdle-induced MST loss in inoculated + girdled trees (I + G) based on each individual tree’s ψ_pd_.


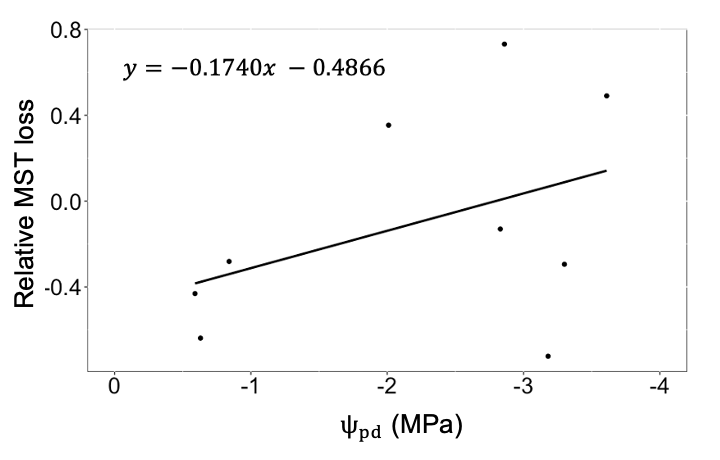


**Fig. S6** Drought depleted inner bark starch concentrations (% dw) but did not affect sugar concentrations (% dw) in *Pinus edulis*. Mean sugar concentration (the sum of sucrose, glucose, fructose) pre-inoculation did not vary among watered and drought-stressed trees (a). Mean starch concentrations of drought-stressed trees pre-inoculation were 90% lower than watered trees (b). Error bars represent +/- SE and gray lowercase letters denote significant differences among drought treatment (*P* < 0.05, ANOVA). Circles represent individual trees. ‘ns’ indicates no significant difference.


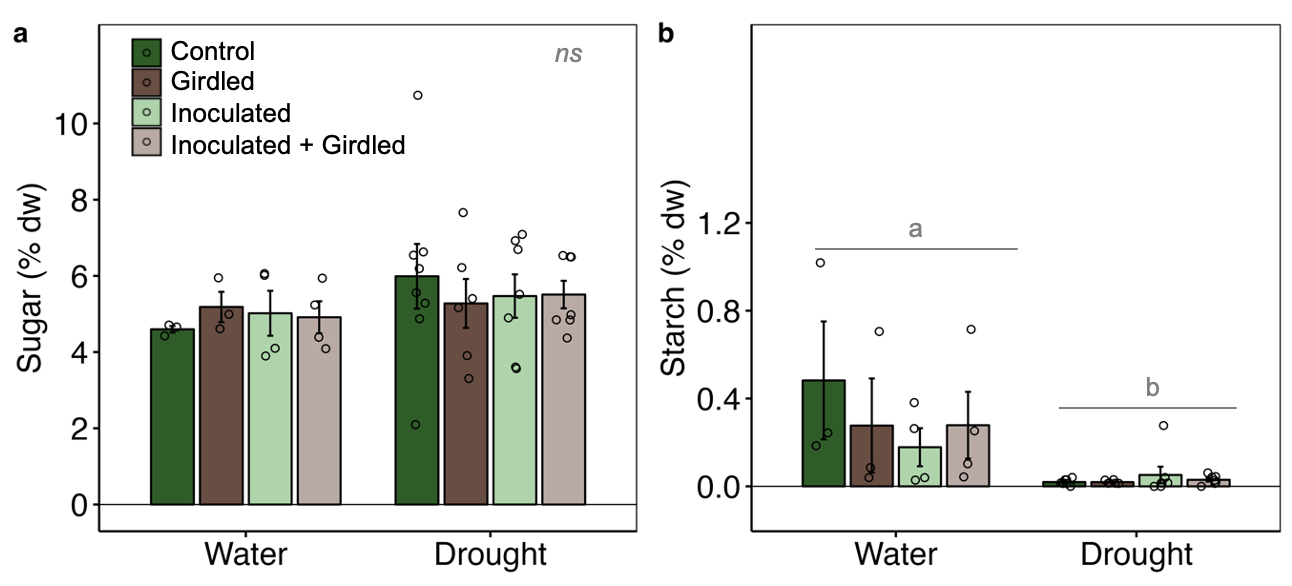


**Fig. S7** Girdle and inoculation treatments had different effects on glucose, fructose, and sucrose in *Pinus edulis*. Glucose + fructose concentrations were reduced relative to the control during the two-week inoculation period (a). Girdle and inoculation treatments did not affect changes in sucrose concentration (% dw) relative to the control during the two-week inoculation period (b). Changes ($\Delta$) in glucose + fructose (a) and sucrose (b) are the difference between post- and pre-inoculation concentrations (measured two weeks apart, see Fig. 1 for detail). Error bars represent +/- SE and gray lowercase letters denote significant differences among group means (*P* < 0.05, ANOVA). Circles represent individual trees.


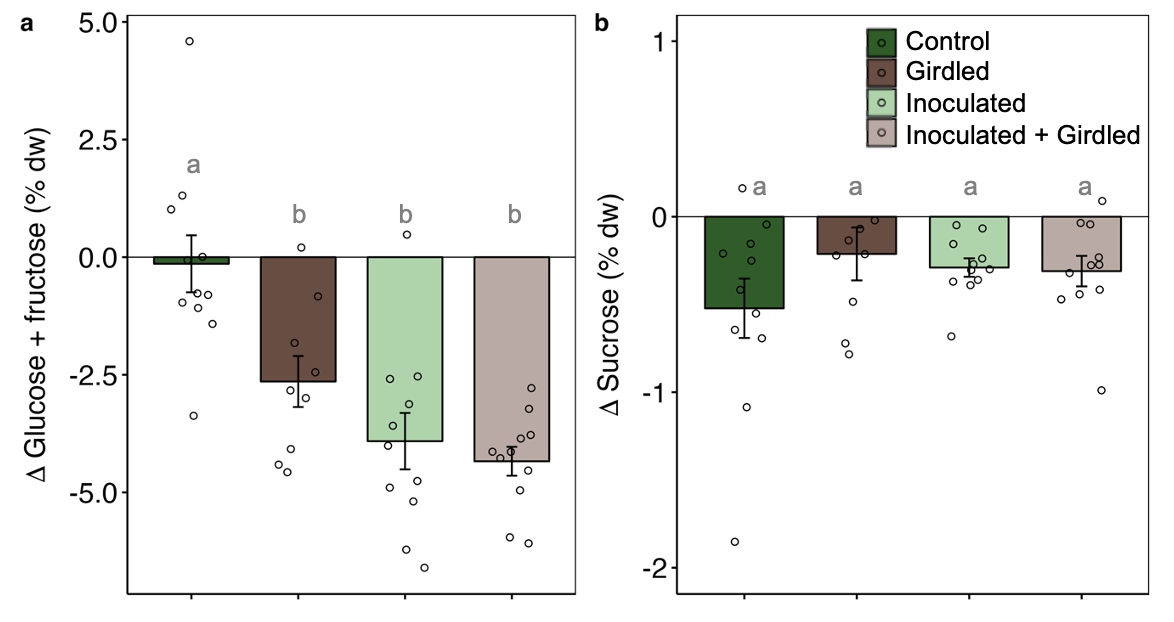


**Fig. S8** Total non-structural carbohydrates (NSC) (% dw) available pre-inoculation were not correlated with induced mono- and sesquiterpene (MST) concentrations ($\Delta$ MST, mg g^-1^) in *Pinus edulis*. Change in MST is the difference between post- and pre-inoculation concentrations. ‘ns’ indicates no significant difference.


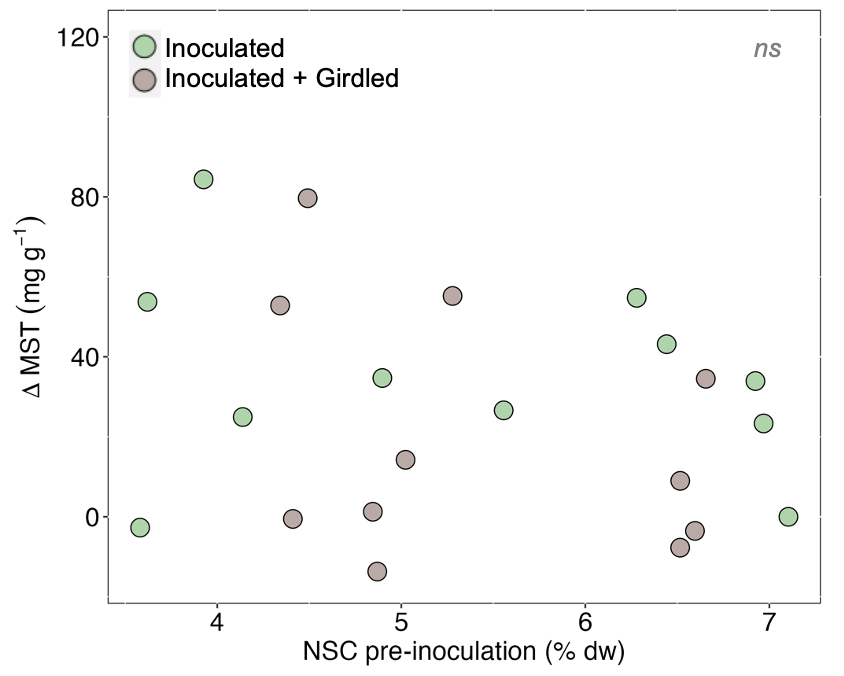


**Table S1.** Individual mono- and sesquiterpene (MST) concentrations (mg g^-1^) [mean (SE)] in response to drought, fungal inoculation, and their interaction (n = 42) in *Pinus edulis*. Significant differences are denoted with lower case letters (*P* < 0.05; Tukey-HSD followed by ANOVA). Compound identification was based upon comparison of retention time and mass spectra with synthetic standards. When synthetic standards were unavailable, the NIST Mass Spectral Search Program (NIST) was used for comparison of mass spectra to determine putative compound identities. The putative compound identity (in parentheses) was used only when the NIST match quality was greater than 80%. MT = unidentified monoterpene; MTO = unidentified oxygenated monoterpene; MTE = unidentified monoterpene ester; SQT = unidentified sesquiterpene; SQTO = unidentified oxygenated sesquiterpene; SQTE = unidentified sesquiterpene ester

|  | **Water**  n = 6 | **Drought**  n = 14 | **Inoculated + Water**  n = 8 | **Inoculated + Drought**  n = 14 |
| --- | --- | --- | --- | --- |
| *Total MST* | 17.11 (5.34) ^a^ | 16.57 (3.49) ^a^ | 55.03 (4.62) ^b^ | 28.23 (3.49) ^a^ |
| *Non-oxygenated monoterpenes* | 10.49 (3.33)^a^ | 10.00 (2.18)^a^ | 32.28 (2.88) ^b^ | 16.86 (2.18) ^a^ |
| MT1 (thujene) | 0 ^a^ | <0.01 ^a^ | 0 ^a^ | 0.02 (0.01) ^a^ |
| (-)-α-pinene | 0.37 (0.12) ^a^ | 0.40 (0.08) ^a^ | 1.47 (0.11) ^b^ | 0.65 (0.08) ^a^ |
| (+)-α-pinene | 6.56 (1.73) ^a^ | 6.59 (1.13) ^a^ | 18.81 (1.50) ^b^ | 9.88 (1.13) ^a^ |
| (-)-camphene | 0.01 (0.01) ^a^ | 0.02 (0.01) ^a^ | 0.09 (0.01) ^a^ | 0.03 (0.01) ^a^ |
| (+)-camphene | 0.03 (0.02) ^a^ | 0.03 (0.01) ^a^ | 0.06 (0.01) ^a^ | 0.05 (0.01) ^a^ |
| β-myrcene | 0.21 (0.19) ^a^ | 0.21 (0.12) ^a^ | 1.27 (0.16) ^b^ | 0.52 (0.12) ^a^ |
| (+)-sabinene | 0.23 (0.52) ^a^ | 0.10 (.034) ^a^ | 0.91 (0.45) ^a^ | 0.88 (0.34) ^a^ |
| (+)-β-pinene | 0.02 (0.01) ^a^ | 0.03 (0.01) ^a^ | 0.12 (0.01) ^b^ | 0.05 (0.01) ^a^ |
| (-)-β-pinene | 0.07 (0.07) ^a^ | 0.11 (0.04) ^a^ | 0.20 (0.06) ^a^ | 0.21 (0.04) ^a^ |
| δ-3-carene | 2.26 (1.27) ^ab^ | 1.86 (0.83) ^a^ | 5.87 (1.10) ^b^ | 2.94 (0.83) ^ab^ |
| trans-β-ocimene and S-(-)-limonene | 0.26 (0.26) ^a^ | 0.32 (0.17) ^a^ | 1.83 (0.17) ^b^ | 0.53 (0.17) ^a^ |
| β-phellandrene | 0.07 (0.07) ^ab^ | 0.06 (0.05) ^a^ | 0.28 (0.06) ^b^ | 0.19 (0.05) ^ab^ |
| γ-terpinene | 0.03 (0.04) ^a^ | 0.02 (0.02) ^a^ | 0.11 (0.03) ^a^ | 0.07 (0.02) ^a^ |
| terpinolene | .036 (0.39) ^a^ | 0.24 (0.25) ^a^ | 1.26 (0.34) ^a^ | 0.81 (0.25) ^a^ |
| *Oxygenated monoterpenes* | 0 ^a^ | 0 ^a^ | 0.01 (0.003) | <0.01 ^a^ |
| Linalool | 0 ^a^ | 0 ^a^ | 0.01 (0.003) ^a^ | <0.01 ^a^ |
| *Monoterpene esters* | 0.18 (0.09) ^a^ | 0.16 (0.06) ^a^ | 0.62 (0.08) ^b^ | 0.34 (0.06) ^a^ |
| MTE1 (4-terpineol acetate) | 0.01 (0.02) ^ab^ | 0.01 (0.01) ^a^ | 0.06 (0.01) ^b^ | 0.03 (0.01) ^ab^ |
| MTE2 (α-terpinyl acetate) | 0.07 (0.05) ^a^ | 0.05 (0.03) ^a^ | 0.25 (0.04) ^b^ | 0.13 (0.03) ^ab^ |
| MTE3 (γ-terpinyl acetate) | 0.01 (0.01) ^a^ | <0.01 ^a^ | 0.02 (0.01) ^a^ | 0.01 (0.01) ^a^ |
| Bornyl acetate | 0.09 (0.03) ^a^ | 0.09 (0.02) ^a^ | 0.29 (0.03) ^b^ | 0.16 (0.02) ^a^ |
| *Sesquiterpenes* | 6.23 (2.04) ^a^ | 6.20 (1.34) ^a^ | 21.02 (1.77) ^b^ | 10.62 (1.34) ^a^ |
| ST1 (rt 35.42) | 0.02 (0.02) ^a^ | 0.02 (0.01) ^a^ | 0.14 (0.01) ^a^ | 0.06 (0.01) ^a^ |
| ST2 (rt 37.50) | 0.01 (0.01) ^a^ | 0.02 (0.01) ^a^ | 0.10 (0.01) ^a^ | 0.03 (0.01) ^a^ |
| ST3 (rt 37.62) | 0.04 (0.01) ^a^ | 0.03 (0.01) ^a^ | 0.02 (0.01) ^a^ | 0.03 (0.01) ^a^ |
| ST4 (α-cadinene) | 0 ^a^ | 0.01 (0.002) ^ab^ | 0.03 (0.003) ^c^ | 0.01 (0.002) ^b^ |
| ST5 (α-bergamotene) | 0.07 (0.06) ^a^ | 0.09 (0.04) ^a^ | 0.32 (0.05) ^b^ | 0.11 (0.04) ^a^ |
| ST6 (α-farnesene) | 0.05 (0.02) ^a^ | 0.03 (0.01) ^a^ | 0.16 (0.02) ^b^ | 0.06 (0.01) ^a^ |
| ST7 ((-)-β-cadinene) | 0.21 (0.11) ^a^ | 0.24 (0.07) ^a^ | 0.98 (0.09) ^b^ | 0.44 (0.07) ^a^ |
| ST8 (trans-β-copaene) | 0.04 (0.02) ^a^ | 0.05 (0.02) ^a^ | 0.20 (0.02) ^b^ | 0.07 (0.02) ^a^ |
| ST9 (cis-β-farnesene) | 0.24 (0.09) ^a^ | 0.16 (0.06) ^a^ | 0.69 (0.08) ^b^ | 0.31 (0.06) ^a^ |
| ST10 (α-cubebene) | 0.10 (0.04) ^a^ | 0.11 (0.02) ^a^ | 0.31 (0.03) ^b^ | 0.18 (0.02) ^a^ |
| ST11 (α-copaene) | 0.62 (0.35) ^a^ | 0.54 (0.23) ^a^ | 2.97 (0.31) ^b^ | 1.30 (0.23) ^a^ |
| ST12 (γ-cadinene) | 0.03 (0.06) ^ab^ | 0.05 (0.04) ^a^ | 0.25 (0.06) ^b^ | 0.09 (0.04) ^ab^ |
| ST13 (rt 37.20) | 0.01 (0.01) ^a^ | 0.01 (0.01) ^a^ | 0.08 (0.01) ^b^ | 0.03 (0.01) ^a^ |
| ST14 (longicyclene) | 0.05 (0.04) ^a^ | 0.06 (0.03) ^a^ | 0.09 (0.04) ^a^ | 0.13 (0.03) ^a^ |
| ST15 (longipinene) | 0.06 (0.03) ^a^ | 0.05 (0.02) ^a^ | 0.24 (0.03) ^b^ | 0.10 (0.02) ^a^ |
| ST16 ((-)-β-elemene) | 0.06 (0.03) ^a^ | 0.05 (0.02) ^a^ | 0.26 (0.03) ^b^ | 0.10 (0.02) ^a^ |
| ST17 ((+)-sativen) | 0.05 (0.03) ^ab^ | 0.06 (0.02) ^a^ | 0.16 (0.03) ^b^ | 0.11 (0.02) ^ab^ |
| β-caryophyllene | 0.09 (0.07) ^a^ | 0.09 (0.05) ^a^ | 0.59 (0.06) ^b^ | 0.22 (0.05) ^a^ |
| (+)-longifolene | 1.22 (0.69) ^a^ | 1.3 (0.45) ^a^ | 1.97 (0.60) ^a^ | 2.34 (0.45) ^a^ |
| ST18 (γ-murrolene) | 0.06 (0.03) ^a^ | 0.08 (0.02) ^a^ | 0.23 (0.02) ^b^ | 0.14 (0.02) ^a^ |
| ST19 (germacreneD) | 3.21 (1.04) ^a^ | 3.12 (0.68) ^a^ | 11.22 (0.90) ^b^ | 4.74 (0.68) ^a^ |
| *Oxygenated sesquiterpenes* | 0.21 (0.12) ^a^ | 0.22 (0.08) ^a^ | 1.12 (0.10) ^b^ | 0.42 (0.08) ^a^ |
| STO1 (rt 33.36) | 0.04 (0.01) ^a^ | 0.03 (0.01) ^a^ | 0.02 (0.01) ^a^ | 0.03 (0.01) ^a^ |
| STO2 (cubebol) | 0.19 (0.10) ^a^ | 0.19 (0.07) ^a^ | 1.03 (0.09) ^b^ | 0.40 (0.07) ^a^ |

**Table S2.** ANOVA analysis of non-structural carbohydrate (NSC) dynamics on mono- and sesquiterpene (MST) induction (mg g^-1^) in *Pinus edulis*.

|  | **All inoculated trees**  (n = 21; inoculated and inoculated + girdled trees across drought treatment) | | **Girdled inoculated trees**  (n = 11; inoculated + girdled trees across drought treatment) | |
| --- | --- | --- | --- | --- |
|  | *F_(1,20)_* | *P* | *F_(1,9)_* | *P* |
| *Pre-inoculation concentration (% dw)* | | | | |
| Total NSC | 1.19 | 0.28 | 0.98 | 0.35 |
| Starch | 0.89 | 0.35 | 1.11 | 0.32 |
| Total Sugar | 1.69 | 0.21 | 1.72 | 0.22 |
| Sucrose | 0.60 | 0.45 | 0.22 | 0.65 |
| Glucose | 1.74 | 0.20 | 1.30 | 0.28 |
| Fructose | 0.71 | 0.40 | 0.46 | 0.51 |
| *Absolute change during 2-week inoculation period (% dw)* | | | | |
| Total NSC | 2.79 | 0.11 | 1.06 | 0.33 |
| Starch | 0.14 | 0.71 | 0.80 | 0.40 |
| Total Sugar | 3.18 | 0.09 | 1.60 | 0.24 |
| Sucrose | 0.73 | 0.40 | 0.30 | 0.60 |
| Glucose | 3.26 | 0.09 | 2.25 | 0.17 |
| Fructose | 2.34 | 0.14 | 0.55 | 0.48 |
| *Relative change during 2-week inoculation period (%)* | | | | |
| Total NSC | 1.89 | 0.18 | 0.09 | 0.78 |
| Starch | 1.89 | 0.18 | 0.06 | 0.82 |
| Total Sugar | 1.87 | 0.19 | 0.08 | 0.79 |
| Sucrose | 0.01 | 0.92 | 0.01 | 0.91 |
| Glucose | 2.22 | 0.22 | 0.06 | 0.81 |
| Fructose | 1.58 | 0.15 | 0.09 | 0.77 |

**Methods S1.** Net photosynthesis and stomatal conductance measurements

Net photosynthesis (Anet, μmol m-2 s-1) and stomatal conductance (gs, mol m-2 s-1) were measured from current-year needles pre-drought, mid-drought, and during the two-week inoculation window (on 8 June 2021, 11 August 2021, and 13 September 2021, respectively).We used a LI-6800 infrared gas-exchange analyzer system (Li-Cor, Lincoln, NE) with a 6 cm2 fluorometer chamber and a reference CO2 concentration of 400 ppm, 1500 μmol m-2 s-1 photosynthetic photon flux density, 25 °C chamber temperature, and 50% relative humidity. Measurements were recorded after steady state gas exchange rates had been maintained for two minutes. Anet was corrected per unit leaf area, and leaf area was estimated using scanned leaf images, ImageJ (imagej.nih.gov), and the R package LeafArea (Katabuchi, 2015).

**Methods S2** Quantification of mono- and sesquiterpenes and non-structural carbohydrates

Inner bark mono- and sesquiterpene (MST) content was quantified using tissue extraction and gas chromatography-mass spectroscopy (GC-MS) protocols described by (Trowbridge *et al.*, 2021). Approximately 100 mg of diced inner bark was weighed into 2 ml Eppendorf tubes and immediately submerged in 1 ml of GC-grade dimethyl chloride (Fisher Scientific, Waltham, MA, USA) with 0.2 $\mu$l ml^-1^ (+)-fenchone (Sigma-Aldrich, Saint Louis, MO) as an internal standard. After being shaken at 125 rpm at 20 °C for 24 hr, we transferred 200 $\mu$l of the supernatant into a 2 ml GC vial lined with a glass insert and capped with a PTFE-lined screw top. The remaining tissue was dried at 70 °C for 48 h to determine terpene concentration per mass of dry tissue (mg g^-1^). Chemical analysis was performed using GC-MS. Two $\mu$l of each sample was injected onto an Agilent Technologies 6890 GC-5973 MS fitted with a Cyclodex-B chiral column (30 m x 250 µm x 0.25 µm: J&W Scientific). Helium was used as the carrier gas at a flow rate of 1.0 ml min^-1^ with a split flow ratio of 30:1. Injector temperature was set to 270 °C and the oven profile consisted of an initial temperature of 40 °C for five minutes followed by a ramp of 3 °C min^-1^ to 200 °C, then a second ramp of 25 °C min^-1^ to 220 °C. Terpene enantiomers were identified by comparing retention times of known standards and mass spectra using the NIST Mass Spectral Library (National Institute of Standards and Technology, Gaithersburg, MD) and OpenLab Chromatography Data System (CDS) software (Agilent Technologies, Wilmington, DE). Concentrations of each compound were calculated using five-point calibration curves with injections of known amounts of pure standards and the internal standard, fenchone. When pure standards were unavailable, standard curves for (+)-$\alpha$-pinene, limonene, bornyl acetate, and caryophyllene were used for monoterpenes, oxygenated monoterpenes, monoterpene esters and sesquiterpenes, respectively. All standards were purchased from Sigma Aldrich (Saint Louis, MO). Chemical concentrations are expressed per mass of dry tissue (mg g ^-1^).

The dry mass content of inner bark starch, sucrose, glucose, and fructose was determined following the standard enzymatic extraction method described by (Landhäusser *et al.*, 2018). After samples were ground with a ball mill (Qiagen Tissuelyser II, Hilden, Germany), soluble sugars were extracted by boiling 30 mg of ground tissue in 1.5 ml of 80% ethanol for 10 min. After a brief centrifugation at 13 000 x g for 1 minute, 200 ul of the supernatant was used to determine soluble sugar concentrations (i.e., glucose, fructose, and sucrose) through enzymatic conversion of sucrose and fructose into glucose by invertase and phosphoglucose isomerase from *Saccharomyces cerevisiae*, respectively (Sigma-Aldrich). The pellet was cleaned two more times with 80% ethanol to remove residual sugar, and the starch remaining in the pellet was broken down to glucose using α-amylase and amyloglucosidase. In both cases, the concentration of glucose was determined photometrically in a 96-well microplate reader (BioTek™ Synergy HT, Winooski, United States) after enzymatic conversion of glucose into glucose-6-phosphate by glucose hexokinase. The concentrations reported are glucose equivalents and reported as % dry weight (% dw).

**Methods S3** Determining the glucose cost required to synthesize 1 g mono- and sesquiterpene.

To determine the glucose cost required to synthesize 1 g terpene, we used an energetic accounting procedure that quantifies synthesis costs related to the carbon skeleton, ATP, and reducing agents (De Vries, 1974; Gershenzon *et al.*, 1993) based on the biochemical pathway for terpene synthesis (Banerjee & Sharkey, 2014). These calculations assume that 1) ATP, NADPH, or NADH produced during terpene biosynthesis are available for reaction within the pathway before external inputs are required; 2) ATP, NADPH, or NADH produced in excess of what is required within the pathway is credited to the final cost; and 3) the glucose cost to generate one mole of ATP, NADPH, and NADH is 0.0278, 0.0856, and 0.0778 mol glucose, respectively (McDermitt & Loomis, 1981; Williams *et al.*, 1987). Mono- and sesquiterpenes are synthesized from the condensation of the five-carbon precursor, isopentenyl diphosphate (IPP) and its isomer dimethylally disphosphate (DMAPP). Therefore, we calculated the glucose cost of a one mole of IPP (5C) and multiplied by either two or three to determine the molar cost of monoterpenes (10C) and sesquiterpenes (15C), respectively. One mole of IPP produced via the methylerythritol phosphate (MEP) pathway (through which most plant mono- and sesquiterpenes are made) requires 1 mol glucose for the carbon skeleton, 3 mol ATP, 3 mol NADPH, and 0 mol NADH (since amounts of NADH produced are greater than those consumed; Banerjee & Sharkey, 2014). This yields a total cost of 1.26, 2.52, and 3.79 moles of glucose per one mole of IPP, monoterpenes, and sesquiterpenes, respectively, and on a gram per gram basis equates to 3.34 g glucose per 1 g mono- or sesquiterpene. The glucose costs estimated here for terpenes synthesized via the MEP pathway are similar to estimates for terpenes synthesized via the mevalonate (MVA) pathway (3.09 g glucose per g terpene (De Vries, 1974) and 3.54 g glucose per g terpene (Gershenzon *et al.*, 1993)).

**Methods S4** Estimates of maximum potential mono- and sesquiterpene induction assuming 100% availability of pre-inoculation non-structural carbohydrate concentrations

We estimated the maximum potential mono- and sesquiterpene (MST) induction assuming that 100% pre-inoculation NSC were available for defense synthesis (as opposed to only 48%). In this case, watered trees induced MST concentrations nearly four times greater than what could be induced based on *de novo* synthesis from NSC availability alone (*P* = 0.002, Tukey-HSD following ANOVA). In contrast, there was no difference between observed and predicted MST induction for drought-stressed trees (*P* = 0.8, Tukey-HSD following ANOVA).

**References**

**Banerjee A, Sharkey TD**. **2014**. Methylerythritol 4-phosphate (MEP) pathway metabolic regulation. *Natural Product Reports* **31**: 1043–1055.

**Gershenzon J, Murtagh GJ, Croteau R**. **1993**. Absence of rapid terpene turnover in several diverse species of terpene-accumulating plants. *Oecologia* **96**: 583–592.

**Landhäusser SM, Chow PS, Dickman LT, Furze ME, Kuhlman I, Schmid S, Wiesenbauer J, Wild B, Gleixner G, Hartmann H, Hoch G, McDowell NG, Richardson AD, Richter A, Adams HD***.* **2018**. Standardized protocols and procedures can precisely and accurately quantify non-structural carbohydrates. *Tree Physiology* **38**: 1764–1778.

**McDermitt D, Loomis R**. **1981**. Elemental composition of biomass and its relation to energy content, growth efficiency, and growth yield. *Annals of Botany* **48**: 275–290.

**Trowbridge AM, Adams HD, Collins A, Dickman LT, Grossiord C, Hofland M, Malone S, Weaver DK, Sevanto S, Stoy PC, McDowell NG***.* **2021**. Hotter droughts alter resource allocation to chemical defenses in piñon pine. *Oecologia* **197**: 921–938.

**De Vries FP**. **1974**. Products, requirements and efficiency of biosynthesis a quantitative approach. *Journal of Theoretical Biology* **45**: 339–377.

**Williams K, Percival F, Merino J, Mooney HA**. **1987**. Estimation of tissue construction cost from heat of combustion and organic nitrogen content. *Plant, Cell & Environment* **10**: 725–734.
